# Hydrogen sulfide‐mediated persulfidation of ERF106 controls pear fruit aroma biosynthesis via disrupting the ERF106‐MYB21‐FAD8 complex

**DOI:** 10.1111/tpj.71129

**Published:** 2026-09-18

**Authors:** Zhuoyan Xu, Kangdi Hu, Longfei Yan, Shuwei Wei, Weisheng Zhao, Huihui Song, Gaifang Yao, Hua Zhang

**Affiliations:** ^1^ School of Food and Biological Engineering Hefei University of Technology Hefei 230009 China; ^2^ Shandong Institute of Pomology Tai'an 271000 China

**Keywords:** pear (*Pyrus ussuriensis*), aroma, hydrogen sulfide (H_2_S), persulfidation, ‘PuERF106‐PuMYB21‐PuFAD8’ molecular module

## Abstract

Hydrogen sulfide (H_2_S) and its mediated protein persulfidation regulate fruit ripening. Fruit aroma, a key quality trait formed during ripening, largely determines consumer preferences. However, the mechanism by which H_2_S modulates aroma biosynthesis in pear remains unclear. This study reveals that H_2_S inhibited aroma biosynthesis in ‘Nanguo’ pear and downregulated the fatty acid desaturase gene *PuFAD8*. Transient overexpression and silencing assays confirmed that *PuFAD8* was required for linoleic acid synthesis, a key aroma precursor. Heterologous overexpression of PuFAD8 in tomato and its knockout in tomato further validated that *PuFAD8* promoted fruit aroma formation, while H_2_S suppressed this function. Transcriptome analysis identified transcription factors PuMYB21 and PuERF106 co‐expressed with *PuFAD8*. Dual‐luciferase and yeast one‐hybrid assays revealed that PuMYB21 bound and activated the *PuFAD8* promoter. Moreover, Y2H, luciferase complementation, pull‐down, and transient expression assays demonstrated that PuERF106 interacted with PuMYB21, synergistically enhancing aroma biosynthesis. Persulfidation at Cys162 of PuERF106 attenuated its interaction with PuMYB21, thereby inhibiting aromatic compound synthesis in pear fruit. Subsequently, the biological function of the PuERF106‐PuMYB21‐PuFAD8 molecular module was validated using stable transgenic pear callus lines and a panel of heterologous and endogenous transgenic tomato lines. This study reveals a novel regulatory mechanism where H_2_S‐mediated persulfidation suppresses pear aroma biosynthesis, providing a theoretical basis for genetic improvement of high‐quality pear.

## Significance Statement

This study elucidates the uncharacterized mechanism whereby H_2_S inhibits pear fruit aroma. It identifies a persulfidation‐mediated ERF106‐MYB21‐FAD8 cascade to advance pear flavor breeding and gaseous signal transduction research.

## INTRODUCTION

Pear is a fruit with tender flesh, a crisp and succulent texture, a characteristic flavor, as well as high nutritional value. The characteristic aroma of pears is primarily determined by a complex mix of volatile organic compounds (VOCs), with esters being the main aromatic compounds that give pears their sweet, fruity scent (Shi, He, et al., 2025). The biosynthesis of aromatic volatiles in fruits is primarily mediated by three major metabolic pathways: fatty acid metabolism, amino acid metabolism, and terpenoid biosynthesis. Fatty acid derivatives represent the primary contributors to fruit flavor quality across most plant species (Wang et al., 2025). Fatty acid catabolism proceeds via two major metabolic routes: the lipoxygenase (LOX) pathway and peroxisomal β‐oxidation. The LOX pathway catalyzes the conversion of linoleic and α‐linolenic acids to aldehydes, alcohols, and their corresponding esters through a sequential enzymatic cascade, whereas β‐oxidation occurs exclusively in peroxisomes, where primarily saturated and monounsaturated fatty acids undergo sequential breakdown via a cyclical series of oxidation, hydration, dehydrogenation, and thiolytic cleavage reactions. The coordinated catalytic activities of key enzymes—including LOX, hydroperoxide lyase (HPL), alcohol dehydrogenase (ADH), alcohol acyltransferase (AAT), and pyruvate decarboxylase (PDC)—synergistically determine the final volatile aroma profile of fruits (Tang et al., 2025). Although hydrogen sulfide (H_2_S) treatment has been shown to modulate flavor‐associated volatile accumulation in ‘Nanguo’ pear fruits (Liu et al., 2014), the underlying molecular mechanisms remain largely uncharacterized. Aroma volatile biosynthesis is critically dependent on the availability of unsaturated fatty acid precursors, and the fatty acid desaturase (FAD) family represents a key class of rate‐limiting enzymes that govern the biosynthesis of these essential precursors (Tang et al., 2025). FADs are integral membrane proteins characterized by three conserved histidine‐rich motifs (His‐boxes) that coordinate the di‐iron catalytic center. Their N‐terminal domains possess either a cytochrome b5 module (endoplasmic reticulum‐localized FAD2/FAD6 isoforms) or a ferredoxin‐dependent reduction system (plastid‐localized FAD3/FAD7/FAD8 isoforms). Double bonds are introduced into oleic acid by these enzymes to generate linoleic acid (C18:2) and α‐linolenic acid (C18:3), which are utilized as the major precursors for LOX pathway‐derived green leaf volatiles, including characteristic C6/C9 aldehydes, alcohols, and esters. In pear fruits, the high expression levels of *PcFAD2* and *PcFAD6* under low‐temperature storage promote the accumulation of linoleic and linolenic acids, thereby enhancing the biosynthesis of ester‐type aroma compounds (Shi, Zhu, et al., 2025). In oil palm, the FAD2/6/7 gene cluster, dominated by *EgFAD2.1*, participates in the regulation of C18 unsaturated fatty acid biosynthesis (Li, Zhou, et al., 2025). Nevertheless, the specific functions of FAD genes during fruit ripening, as well as their regulatory networks with upstream transcription factors, remain to be further elucidated.

The synthesis of fruit aroma involves a complex regulatory network of transcription factors, among which MYB, ERF, NAC, WRKY, and other families play key roles in modulating aroma formation. Transcription factors often function through synergistic interactions to regulate aroma biosynthetic pathways. In strawberries, a transcriptional complex formed by FaERF#9 and FaMYB98 directly bound to and activated the promoter of the quinone reductase gene *FaQR*, whereby the biosynthesis of characteristic aroma compounds was synergistically regulated (Zhang et al., 2018). In pear fruit, PuERF13 acted synergistically with the Dof transcription factor PuDof2.5 to activate the promoter of the alcohol acyltransferase gene *PuAAT1*, promoting ester aroma production during low‐temperature storage (Li et al., 2023). In peaches, PpNAC1 not only activated *PpAAT1* to enhance ester biosynthesis but also synergistically regulated *PpFAD3‐1* to influence the supply of aroma precursors (Song et al., 2021). In bananas, the MaMYB69‐MaERF55‐MabZIP5 module synergistically regulated multiple aspects of fruit quality (Liu et al., 2026). Alternatively, transcription factors can independently regulate aroma metabolic pathways. In strawberries, FaMYB11 promoted the accumulation of C6 aldehyde and ester volatiles by directly activating the promoter of the lipoxygenase gene *FaLOX5* (Lu et al., 2021). In tomatoes, SlMYB75 directly activated the promoters of key aroma genes such as LOXC, thereby enhancing the accumulation of terpenoid, aldehyde, and phenylpropanoid volatiles (Jian et al., 2019). In melon, CmERFI‐2 directly bound to and repressed the promoter of *CmMYB44*, forming an ERF‐MYB transcriptional cascade that modulated fruit quality (Gao et al., 2023). In cardamom, AP2/ERF1 positively regulated sesquiterpene biosynthesis (e.g., β‐elemene), whereas NAC6 controlled monoterpene production (e.g., β‐pinene), illustrating the sophisticated hierarchical and cooperative regulatory mechanisms underlying fruit aroma formation (Ma et al., 2024). Nevertheless, the precise molecular mechanisms by which direct physical interactions between MYB and ERF transcription factors regulate volatile aroma biosynthesis in pear fruits remain largely elusive, underscoring the critical need to decipher how these transcriptional regulators synergistically modulate pear aroma production.

Hydrogen sulfide (H_2_S) has emerged as a key gaseous signaling molecule that regulates diverse plant physiological processes, including postharvest fruit ripening, seed germination, root system development, and stomatal aperture control (Jaiswal et al., 2024). H_2_S exerts its biological functions primarily through reversible covalent modification of reactive cysteine thiol groups (‐SH) on target proteins, forming persulfide (‐SSH) moieties—a post‐translational modification known as protein persulfidation (Corpas, 2025). This reversible modification dynamically alters protein three‐dimensional structure, catalytic activity, intracellular stability, and protein–protein interaction networks. In tomato fruits, H_2_S‐mediated persulfidation of SlERF.D2 repressed the expression of ethylene biosynthesis and signaling genes, leading to delayed postharvest ripening (Sun, Zhang, et al., 2025). H_2_S can also indirectly regulate transcription factor stability by modifying E3 ubiquitin ligases via persulfidation, which in turn modulates fruit ripening kinetics (Sun et al., 2023). Additionally, persulfidation of SlERF.D3 enhanced its transcriptional activation activity while simultaneously inhibiting its ubiquitination‐dependent proteasomal degradation, acting synergistically to delay fruit ripening (Hu, Geng, et al., 2025). In *Brassica rapa*, H_2_S‐induced persulfidation of BraFLC reduced its DNA‐binding affinity, altered the alternative splicing patterns of flowering‐related genes, and accelerated floral transition (Jin, 2026). Beyond developmental processes, H_2_S‐mediated thiol modifications also regulate plant immune responses and autophagic degradation pathways (Hu, Qin, et al., 2025). Collectively, H_2_S‐mediated protein persulfidation modifications are integral to multiple facets of plant development; however, whether such post‐translational modifications govern the accumulation of aroma compounds in pear fruit remains largely unexplored.

Here, we investigated the molecular mechanism by which hydrogen sulfide (H_2_S) regulates volatile aroma biosynthesis in ‘Nanguo’ pear fruits. We integrated metabolomic, transcriptomic, and bioinformatic analyses of H_2_S‐treated ‘Nanguo’ pear fruits to identify *PuFAD8* as a key rate‐limiting gene involved in aroma regulation. While *PuFAD8* expression is transcriptionally responsive to H_2_S signaling, the PuFAD8 protein itself does not undergo direct persulfidation modification. Instead, two upstream transcription factors, *PuMYB21* and *PuERF106*, form a heteromeric transcriptional activation complex that directly binds to the *PuFAD8* promoter and drives its expression, thereby regulating the biosynthesis of unsaturated fatty acids and downstream aroma volatiles. H_2_S negatively modulates this regulatory cascade via site‐specific persulfidation of PuERF106 at the Cys162 residue, which disrupts the formation and transcriptional activity of the PuMYB21‐PuERF106 complex. Functional validation experiments using overexpression and CRISPR/Cas9‐mediated knockout of the tomato ortholog *SlFAD8* further confirmed these findings, yielding results consistent with our observations in pear fruits. Collectively, our results uncovered a previously unrecognized H_2_S‐mediated post‐translational regulatory mechanism that controls fatty acid‐derived aroma biosynthesis in ‘Nanguo’ pear, providing a valuable theoretical foundation for molecular breeding to enhance fruit flavor quality.

## RESULTS

### Exogenous H_2_S treatment modulates fatty acid metabolic pathways in ‘Nanguo’ pear fruits

**Figure 1:**
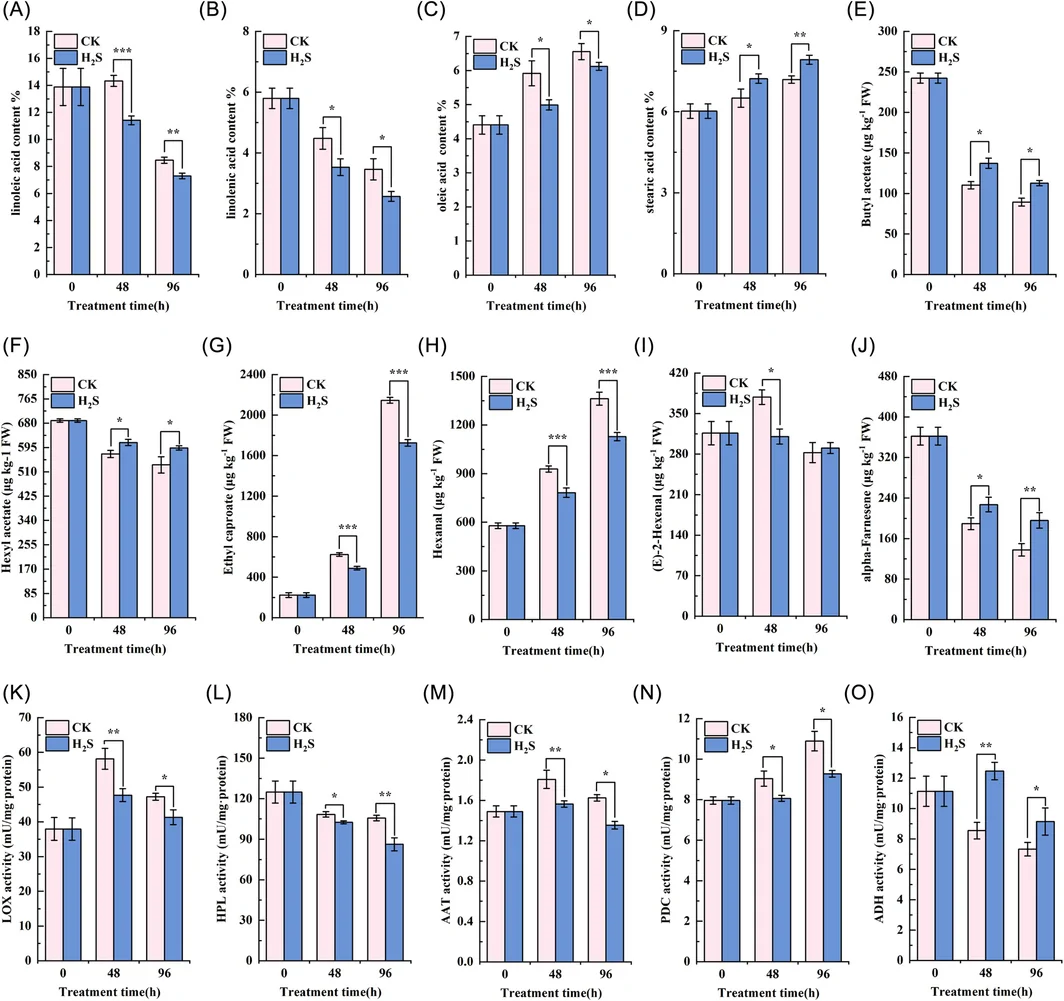
Effects of H_2_S fumigation on fatty acid metabolism and related volatile aroma compounds in ‘Nanguo’ pear. (A–D) Changes in the contents of α‐linolenic acid, linoleic acid, stearic acid, and oleic acid in ‘Nanguo’ pear fruits at 0, 48, and 96 h after H_2_S fumigation. Linolenic acid (A), linoleic acid (B), oleic acid (C), stearic acid (D). (E–J) Changes in the contents of Butyl acetate, Hexyl acetate, Ethyl hexanoate, Hexanal, (E)‐2‐Hexenal, and αlpha‐farnesene in ‘Nanguo’ pear fruits at 0, 48, and 96 h after H_2_S fumigation. Butyl acetate (E), Hexyl acetate (F), Ethyl hexanoate (G), Hexanal (H), (E)‐2‐Hexenal (I), αlpha‐Farnesene (J). (K–O) Enzyme activities of lipoxygenase (LOX), hydroperoxide lyase (HPL), alcohol acyltransferase (AAT), alcohol dehydrogenase (ADH), and pyruvate decarboxylase (PDC) in ‘Nanguo’ pear fruit at 0, 48, and 96 h after H_2_S fumigation. LOX activity (K), HPL activity (L), AAT activity (M), PDC activity (N), ADH activity (O). All data are presented as mean ± standard deviation (SD) of at least three biological replicates. Statistical significance was determined by Student's *t*‐test (*P < 0.05, **P < 0.01, ***P < 0.001).

Guided by the metabolic pathways of aroma compounds in higher plants (Figure S1A), gas chromatography–mass spectrometry (GC–MS) was utilized to profile the levels of aroma compounds and fatty acids in hydrogen sulfide (H_2_S)‐treated ‘Nanguo’ pear (*Pyrus ussuriensis* Maxim. cv. Nanguoli) fruits, revealing that exogenous H_2_S application significantly repressed the biosynthesis of most aroma compounds and pigment precursors. Principal component analysis (PCA) (Figure S1B) demonstrated that H_2_S treatment markedly reduced aroma components in pear fruits at 48 and 96 h relative to the control. Fatty acid compositions followed the above trends. Time‐dependent declines were found in linolenic and linoleic acids, with a stronger reduction of both acids in H_2_S‐exposed fruits. Oleic acid and stearic acid were slightly accumulated (Figure 1A–D). Profiling of representative volatile aroma compounds derived from fatty acid metabolic pathways revealed that H_2_S treatment significantly decreased the levels of ethyl hexanoate, hexanal, and (E)‐2‐hexenal, and suppressed the biosynthesis of butyl acetate and alpha‐fenchene relative to the control group (Figure 1E–J). H_2_S treatment reduced the activities of LOX, HPL, AAT, PDC, and ADH (Figure 1K–O).

### Identification of *PuFAD8* through transcriptome sequencing of H_2_S‐treated ‘Nanguo’ pear fruits

**Figure 2:**
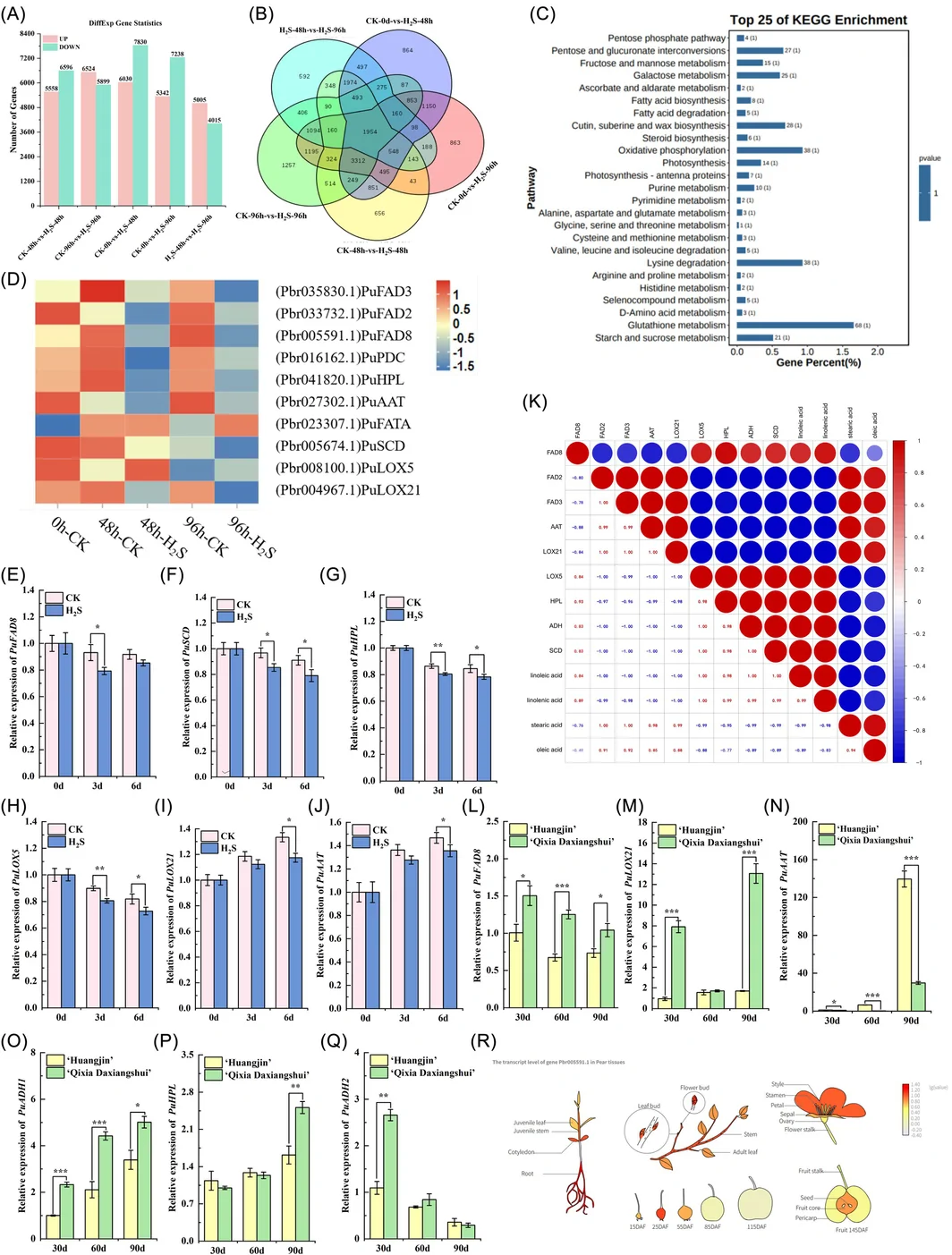
*PuFAD8* is potentially involved in aroma and fatty acid biosynthesis during the early ripening stage of ‘Nanguo’ pear. (A–C) Transcriptomic profiling of differentially expressed genes (DEGs) in ‘Nanguo’ pear fruits in response to H_2_S fumigation. Statistical summary of DEGs (A), Venn diagram analysis of DEGs across different comparison groups (B), and KEGG pathway enrichment analysis of all identified DEGs (C). (D) Heatmap visualization of candidate fatty acid desaturase (FAD) genes involved in the fatty acid metabolic pathway. (E–J) RT‐qPCR analysis of transcript levels for PuFADs and aroma biosynthesis‐related genes in ‘Nanguo’ pear fruit following H_2_S treatment. *PuFAD8* (E), *PuSCD* (F), *PuHPL* (G), *PuLOX5* (H), *PuLOX21* (I), and *PuAAT* (J). (K) Correlation analysis between the expression levels of aroma biosynthesis‐related genes, enzyme activities, and fatty acid contents in ‘Nanguo’ pear fruit after H_2_S treatment. (L–Q) RT‐qPCR analysis was performed to determine the transcript levels of PuFADs and aroma biosynthesis‐related genes in ‘Huangjin’ pear and ‘Qixia Daxiangshui’ pear fruits. *PuFAD8* (L), *PuLOX21* (M), *PuAAT* (N), *PuADH1* (O), *PuHPL* (P), *PuADH2* (Q). (R) Prediction of the spatiotemporal expression pattern of *PuFAD8*. All bar graph data are presented as mean ± standard deviation (SD) of at least three biological replicates. Statistical significance was evaluated by Student's *t*‐test (*P < 0.05, **P < 0.01, ***P < 0.001).

Transcriptome profiles of H_2_S‐treated ‘Nanguo’ pear fruits were comprehensively investigated to screen candidate genes regulating fatty acid‐derived volatile aroma metabolites. As illustrated in Figure 2(A), differentially expressed genes (DEGs) were profiled in pear fruit samples at 0, 48, and 96 h post‐H_2_S exposure. Compared with untreated controls, 6030 genes were significantly upregulated and 7830 downregulated at 48 h post‐H_2_S treatment, while 5342 upregulated and 7238 downregulated DEGs were identified at 96 h, with Venn diagram analysis revealing 1954 overlapping genes common to both time points (Figure 2B). KEGG functional enrichment analysis revealed that 8% of these overlapping DEGs were primarily overrepresented in fatty acid biosynthesis, while an additional 5% were assigned to the fatty acid degradation pathway (Figure 2C).

Following this, multiple differentially expressed genes involved in fatty acid metabolic pathways were prioritized as key regulatory candidates, among which three PuFAD family members were predicted to mediate the biosynthesis of fatty acid‐derived aroma volatiles in ‘Nanguo’ pear fruits (Figure 2D). To elucidate the regulatory mechanism underlying H_2_S‐mediated fatty acid metabolic reprogramming, quantitative real‐time PCR (qRT‐PCR) was performed to profile the expression profiles of key aroma‐associated structural genes, revealing that the transcript levels of *PuFAD8*, *PuSCD*, *PuHPL*, and *PuLOX5* were significantly downregulated at 0, 3, and 6 days post‐H_2_S exposure (Figure 2E–H). In contrast, exogenous H_2_S application markedly upregulated the transcriptional levels of *PuFAD2*, *PuFAD3*, *PuLOX21*, and *PuAAT* throughout the sampling period (Figure 2I–J; Figure S2A,B).

Correlation analysis further identified functional regulatory links between candidate genes and H_2_S‐mediated metabolic changes. *PuFAD8* showed strong positive correlations with *PuLOX5*, *PuSCD*, and *PuADH1* expression, HPL activity, and linoleic and linolenic acid contents, while *PuFAD2* and *PuFAD3* were positively correlated with LOX and AAT activities and linoleic acid accumulation (Figure 2K). Comparative expression analysis was conducted in two pear cultivars with contrasting aroma intensities. All core aroma biosynthetic genes were significantly upregulated in the highly aromatic ‘Qixia Daxiangshui’ compared to the low‐fragrance ‘Huangjin’ across postharvest ripening stages. During postharvest storage, *PuFAD8* expression was gradually downregulated in both cultivars (Figure 2L), whereas *PuFAD2* and *PuFAD3* were progressively upregulated (Figure S2C,D). Downstream aroma genes were coordinately activated during fruit senescence (Figure 2M–P), and *PuADH2* expression was highly synchronized with *PuFAD8* (Figure 2Q). Tissue and developmental expression analyses confirmed that *PuFAD8* is most highly expressed at early fruit developmental stages, with its transcription declining as fruit maturation proceeds (Figure 2R). Given that earlier studies have established *PuFAD2* and *PuFAD3* as principal regulators of aroma biosynthesis during late fruit ripening (Liu, Qin, et al., 2024), the functional characterization of *PuFAD8* was prioritized as the core research objective in this study.

### *PuFAD8* contributes to the biosynthesis of fruit aroma volatiles

To elucidate the evolutionary position and potential contribution of *PuFAD8* to fatty acid‐derived volatile aroma metabolism, a phylogenetic tree was constructed (Figure S3A), which placed *PuFAD8* in a clade with the ω‐6 desaturase *AtFAD6*, reflecting close evolutionary homology (Falcone et al., 1994). As *AtFAD6* has been functionally validated as a critical regulator of plant fatty acid biosynthesis, *PuFAD8* is predicted to similarly mediate fatty acid synthesis during early fruit development; all FAD family members share three highly conserved histidine motifs (the canonical HXXXH, HXXHH, and QXXHH sequences, Figure S3B), and multiple sequence alignments confirmed that *PuFAD8* exhibits identical conserved structural features to other plant ER‐type FAD proteins.

**Figure 3:**
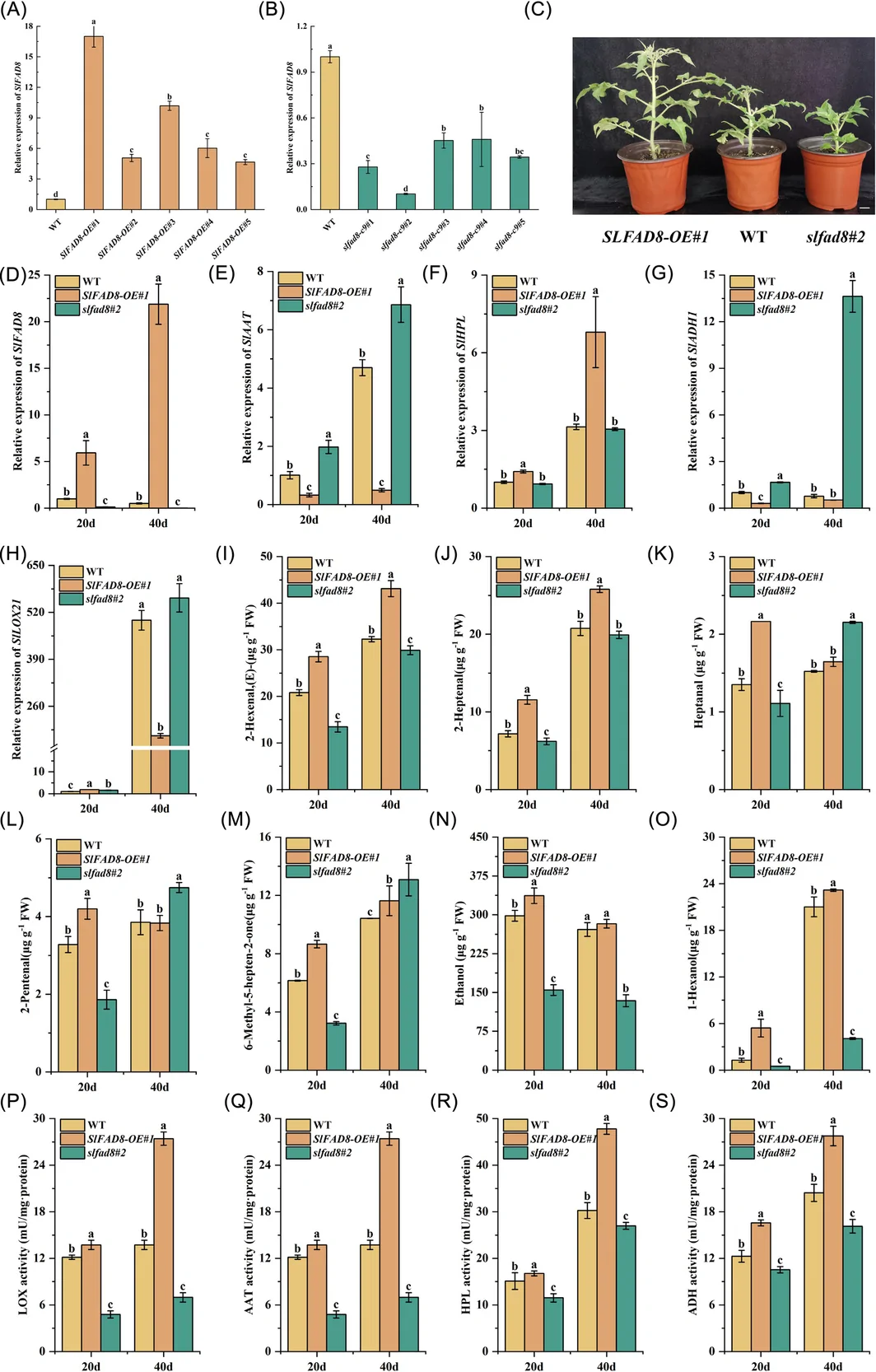
Generation and functional analysis of transgenic tomato lines with altered *SlFAD8* expression. (A, B) RT‐qPCR validation of *SlFAD8* transcript levels in independent transgenic lines: (A) *SlFAD8‐OE* lines, (B) s*lfad8* mutant lines, relative to WT. (C) Phenotypic comparison of wild‐type (WT), *SlFAD8*‐overexpressing (*SlFAD8‐OE*), and s*lfad8* mutant tomato plants. Scale bars = 1 cm. (D–H) Expression profiling of *SlFAD8* and key aroma biosynthetic genes in tomato fruit at different developmental stages: *SlFAD8* (D), *SlAAT* (E), *SlHPL* (F), *SlADH* (G), *SlLOX21* (H). (I–O) Quantitative analysis of volatile aroma compounds in ripe tomato fruits: 2‐Hexenal (E)–(I), 2‐Heptenal (J), Heptanal (K), 2‐Pentenal (L), 6‐Methyl‐5‐hepten‐2‐one (M), Ethanol (N), 1‐Hexanol (O). (P–S) Enzymatic activity assays of key lipoxygenase pathway enzymes involved in aroma formation: LOX (P), AAT (Q), HPL (R), ADH (S). All bar graph data are presented as mean ± standard deviation (SD) from at least three independent biological replicates. Different letters indicate significant differences between samples (*P* < 0.05, Duncan's test).

Functional validation of *PuFAD8* was performed via transient overexpression and RNAi silencing in early‐maturing pear fruits (Figure S4A–I). *PuFAD8* overexpression significantly upregulated downstream aroma biosynthetic genes (*PuHPL*, *PuLOX5*, *PuAAT*, and *PuADH1*), while exogenous H_2_S treatment exerted the opposite effect. Consistently, the expression of these genes was significantly reduced by *PuFAD8* knockdown. Given the technical limitations of stable pear transformation, the tomato ortholog *SlFAD8* (*Solyc08g063090.2.1*, 60.85% amino acid identity) was used for functional validation of *PuFAD8*. *SlFAD8*‐overexpressing lines and T_2_‐generation homozygous *slfad8* knockout mutants were generated and confirmed by qRT‐PCR (Figure 3A–C; Figure S5). The highest‐expressing overexpression line and lowest‐expressing knockout line were selected for further analysis. Transcriptional analysis revealed that key aroma genes were significantly downregulated in *SlFAD8‐OE* fruits at 40 days post‐anthesis compared with WT and *slfad8* mutants (Figure 3B–H). Volatile metabolite profiling showed distinct patterns: C6/C7 aldehydes and alcohols were elevated in *SlFAD8‐OE* fruits, while other volatiles accumulated preferentially in *slfad8* mutants at the late ripening stage (Figure 3I–O). Activities of core aroma biosynthetic enzymes were also determined among the three genotypes (Figure 3P–S).

### Exogenous H_2_S indirectly regulates *PuFAD8* to modulate fatty acid‐derived volatile aroma formation in pear fruits

**Figure 4:**
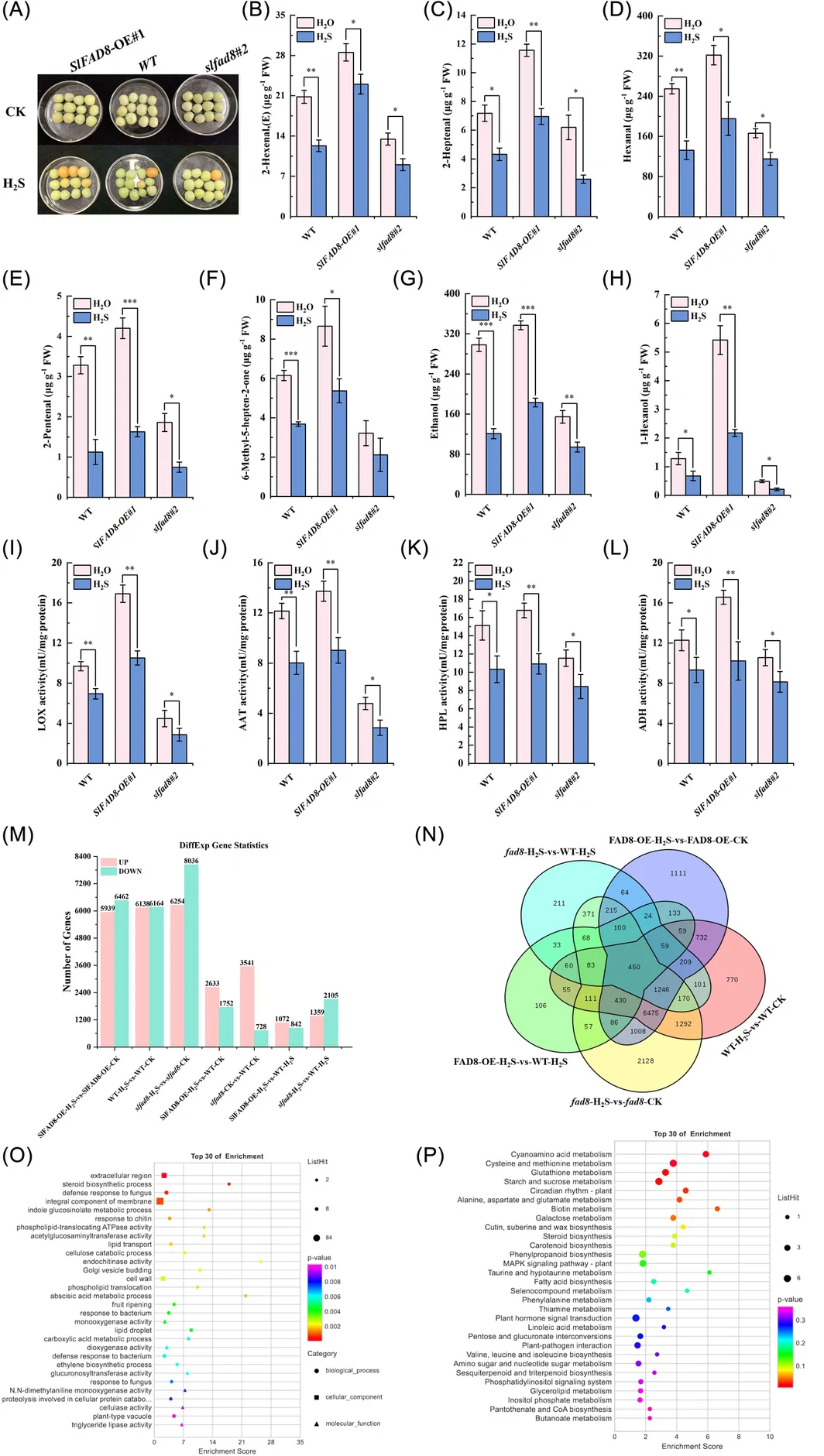
Indirect modulation of *SlFAD8* by H_2_S regulates tomato fruit aroma biosynthesis. (A) Phenotypic observation of homozygous *SlFAD8* tomato fruits at 3 days post H_2_S treatment (dpt). Scale bars = 1 cm. (B–H) Quantification of volatile aroma‐related compound contents in *SlFAD8* tomato fruits at 3 days after H_2_S treatment: 2‐Hexenal (B), 2‐Heptenal (C), Heptanal (D), 2‐Pentenal (E), 6‐Methyl‐5‐hepten‐2‐one (F), Ethanol (G), and 1‐Hexanol (H). (I–L) Determination of enzyme activities associated with aroma compound biosynthesis in *SlFAD8* tomato fruits at 3 days post H_2_S treatment: LOX (I), AAT (J), HPL (K), and ADH (L). (M–P) Differential gene expression analysis using H_2_S‐treated SlFAD8‐overexpressing (*SlFAD8*‐OE), *slfad8* mutant, and wild‐type (WT) tomato fruits as experimental materials: Venn diagram showing the overlap of differentially expressed genes (DEGs) (M), DEG expression profile (N), Gene Ontology (GO) enrichment analysis of DEGs (O), and Kyoto Encyclopedia of Genes and Genomes (KEGG) enrichment analysis of DEGs (P). All data in bar graphs are presented as mean ± standard deviation (SD), with at least three independent biological replicates. Statistical significance was evaluated using Student's *t*‐test: *P < 0.05, **P < 0.01, ***P < 0.001.

To explore the molecular basis of H_2_S regulation of FAD8‐mediated aroma biosynthesis, mature green WT, *SlFAD8‐OE*, and *slfad8* tomato fruits were treated with H_2_S or water daily for 3 days; H_2_S significantly delayed pigment accumulation in all genotypes, with the strongest effect in *SlFAD8‐OE* (Figure 4A). GC–MS profiling confirmed that H_2_S globally reduced volatile aroma compounds in all tomato genotypes (Figure 4B–H), demonstrating H_2_S negatively regulated aroma biosynthesis (most dramatic changes in *SlFAD8‐OE*), and showing core aroma enzymes (LOX, AAT, HPL, ADH) were diminished in H_2_S‐treated *SlFAD8‐OE* and *slfad8* fruits (Figure 4I–L).

Transcriptomic profiling of identically treated tomato fruits yielded DEGs across groups (Figure 4M); Venn analysis showed H_2_S induced 12 401, 12 302 and 14 290 DEGs in *SlFAD8‐OE*, WT and *slfad8* tomatoes, respectively (Figure 4N). Global transcriptional reprogramming was evident in all tomato genotypes upon exogenous H_2_S application; relative to their respective controls, H_2_S induced 5939 upregulated and 6462 downregulated DEGs in *SlFAD8‐OE* lines (Figure S6A), 6138 upregulated and 6138 downregulated DEGs in WT fruits (Figure S6B), and 6254 upregulated and 8036 downregulated DEGs in *slfad8* mutants (Figure S6C). GO enrichment of shared DEGs across three genotypes revealed core enrichment in fatty acid biosynthesis, aromatic amino acid metabolism, and fatty acid metabolism (Figure 4O), while KEGG analysis showed significant clustering in cysteine and methionine metabolism (Figure 4P). A modified biotin‐switch assay detected no persulfidation modification of the *PuFAD8* protein after H_2_S treatment (the faint band in the figure corresponds to non‐specific signal) (Figure S7A), confirming that H_2_S regulated *PuFAD8*‐mediated aroma biosynthesis through an indirect rather than direct mechanism.

### *PuMYB21* directly binds the *PuFAD8* promoter and positively modulates fatty acid‐derived volatile aroma biosynthesis in pear fruits

**Figure 5:**
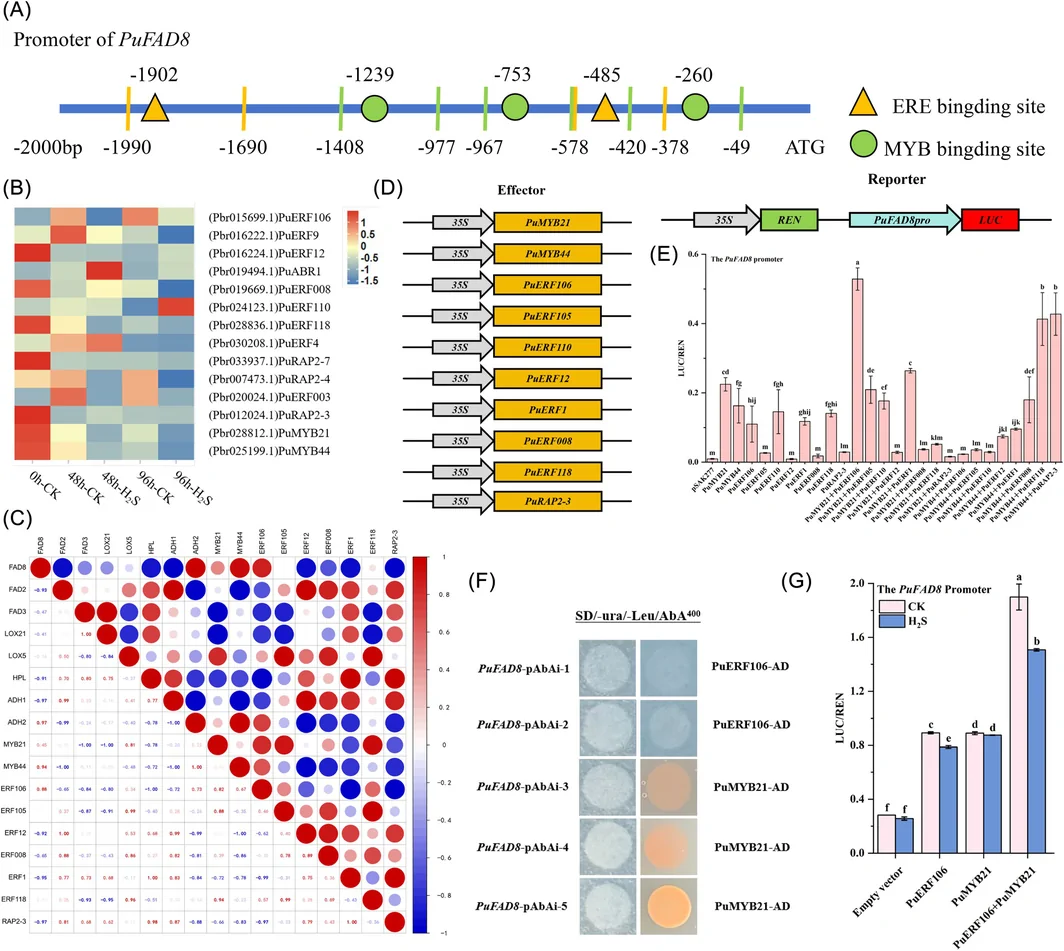
PuMYB21 binds to the *PuFAD8* promoter and mediates its transcriptional activation. (A) *Cis*‐acting element analysis of the *PuFAD8* promoter was performed to identify potential regulatory motifs. (B) Candidate transcription factor (TF) selection: MYB and ERF family members with expression patterns correlating with *PuFAD8* were identified from transcriptomic data, and their transcript levels were quantified. (C) Correlation analysis between *PuFAD8* and the candidate MYB/ERF TFs (identified in B) was conducted to validate co‐expression patterns. (D) Dual‐luciferase reporter (DLR) assay for TF activation: Schematic representation of effector constructs (expressing *PuMYB21*, *PuMYB44*, *PuERF106*, *PuERF105*, *PuERF110*, *PuERF12*, *PuERF1*, *PuERF008*, *PuERF118*, or *PuRAP2‐3*) and the reporter construct containing the *PuFAD8* promoter driving firefly luciferase (LUC), with Renilla luciferase (REN) as an internal control. (E) Validation of *PuFAD8* transcriptional activation by candidate TFs via DLR assay. Data are presented as mean ± standard deviation (SD). Asterisks indicate significant differences (Student's *t*‐test, *P* < 0.05). (F) Yeast one‐hybrid (Y1H) analysis demonstrated that PuMYB21 binds specifically to the *PuFAD8*‐S5 promoter fragment, but not to the S3 or S4 regions; no binding of *PuERF106* to any *PuFAD8* promoter fragment was detected. (G) DLR assay under H_2_O/H_2_S treatment: The combined effect of *PuERF106* and *PuMYB21* on *PuFAD8* transcriptional activation was evaluated. Data are presented as mean ± SD. Different lowercase letters above bars indicate significant differences (one‐way ANOVA followed by Tukey's HSD test, *P* < 0.05).

To identify upstream transcriptional regulators that directly target the *PuFAD8* promoter, we analyzed the 2‐kb genomic sequence upstream of the *PuFAD8* translation start site and predicted conserved MYB and ERF family cis‐acting regulatory elements within this region (Figure 5A). By integrating transcriptome datasets generated from H_2_S‐treated ‘Nanguo’ pear fruits, we identified two MYB and twelve ERF transcription factors exhibiting significant co‐expression patterns with *PuFAD8*. We subsequently profiled their temporal expression dynamics across multiple fruit developmental stages in two pear cultivars with divergent aromatic phenotypes (Figure 5B), followed by Pearson correlation analysis to quantify their expression relationships (Figure 5C). *PuMYB21* transcript levels showed highly significant positive correlations with several ERF family members, among which *PuERF106* exhibited the highest correlation coefficient (*R* = 0.957) and was also strongly correlated with *PuFAD8* (*R* = 0.923). Remarkably, *PuFAD8* and *PuERF106* displayed the strongest expression correlation among all analyzed gene pairs, with a Pearson coefficient reaching 0.993.

Dual‐luciferase reporter assays confirmed that co‐expression of MYB and ERF transcription factors activated the *PuFAD8* promoter more strongly than individual overexpression, with the PuMYB21‐PuERF106 combination exhibiting the highest transactivation activity among all tested pairs (Figure 5D,E). We hypothesized that *PuMYB21* and *PuERF106* synergistically regulate *PuFAD8* via protein interaction; Y1H assays using five truncated *PuFAD8* promoter baits (400 ng ml^−1^ AbA for self‐activation suppression) confirmed that *PuMYB21* directly bound the third promoter fragment to activate transcription, whereas *PuERF106* showed no direct binding affinity (Figure 5F). Additional dual‐luciferase assays confirmed that *PuMYB21‐PuERF106* co‐expression strongly activated *PuFAD8* transcription via heterodimer formation (Figure 5G), while exogenous H_2_S significantly reduced the activity of *PuERF106*‐containing complexes.

### PuMYB21‐PuERF106 heterodimeric complex regulates pear fruit volatile aroma formation

**Figure 6:**
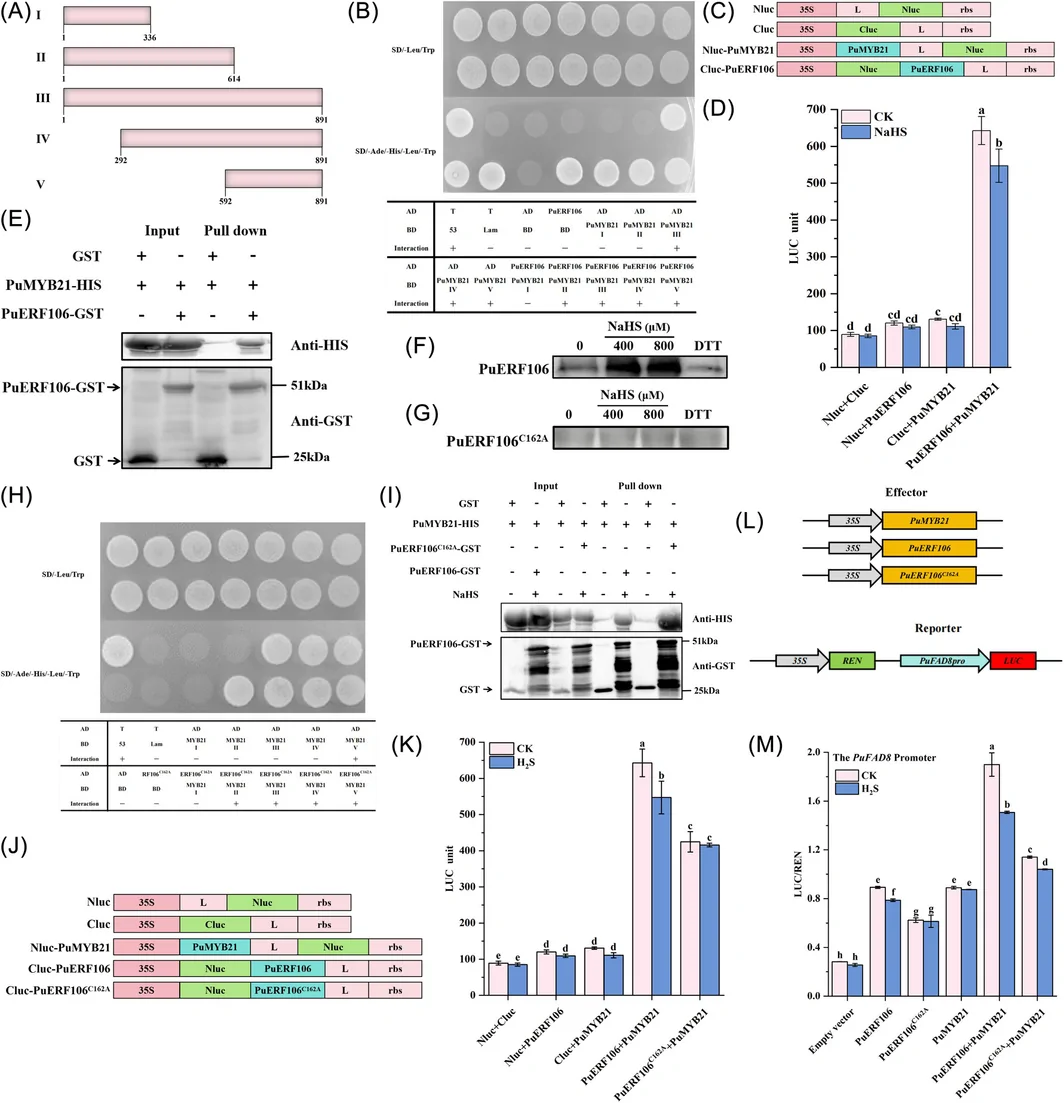
Physical interaction between PuERF106 and PuMYB21. (A) Schematic illustration showing five key amino acid residues (I‐V) of the PuMYB21 protein. (B) Y2H assay verifying the physical interaction between PuMYB21 and PuERF106. AD‐empty + BK‐empty and AD‐empty + BK53 were used as negative controls; AD + BK‐Lam served as the positive control. (C) Schematic structure of the split luciferase vectors Nluc and Cluc used for protein interaction detection. (D) Firefly Luciferase Complementation Imaging (LCI) Assay validating the interaction strength between PuMYB21 and PuERF106 under H_2_O and H_2_S treatments. Sodium hydrosulfide (NaHS) was applied as an exogenous H_2_S donor. Data are shown as mean ± standard deviation (SD). (E) *In vitro* pull‐down assay confirming the direct protein–protein interaction between PuERF106 and PuMYB21. (F, G) Recombinant PuERF106 and PuERF106^C162A^ proteins were subjected to disulfide modification via a modified biotin switch assay. Proteins were incubated with NaHS at graded concentrations (0, 400, and 800 μM) or 1 mM DTT, and disulfide‐modified PuERF106/PuERF106^C162A^ was detected by Western blot analysis. (H) Y2H assay was performed to verify the physical interaction between PuMYB21 and PuERF106. AD+BK and AD+BK53 served as negative controls, while AD+BK‐Lam was used as the positive control. (I) *In vitro* pull‐down assay examining the interaction between H_2_O/H_2_S‐treated PuERF106/PuERF106^C162A^ and PuMYB21. (J) Schematic representation of the Nluc and Cluc fusion vectors used for LCI assays. (K) LCI assay validating the *in vivo* interaction between PuMYB21 and PuERF106 under H_2_O or H_2_S treatment. Data are shown as mean ± SD of three biological replicates. (L) Schematic diagram of effector (transcription factors: PuMYB21, PuERF106, PuERF106^C162A^) and reporter (*PuFAD8* promoter driving luciferase) constructs used in DLR assays. REN: Renilla luciferase (internal control); LUC: Firefly luciferase (reporter). DLR assay assessing the transcriptional activation of *PuFAD8* by PuMYB21, PuERF106, and PuERF106^C162A^. (M) DLR assay evaluating the combinatorial activation of *PuFAD8* by PuERF106/PuERF106^C162A^ and PuMYB21 under H_2_O or H_2_S treatment. Data are presented as mean ± SD. Different letters above bars indicate significant differences (one‐way ANOVA followed by Duncan's multiple range test, *P* < 0.05).

To further characterize the physical interaction between PuMYB21 and PuERF106, truncated variants of PuMYB21 were constructed according to its conserved functional domains, including PuMYB21^1‐336^, PuMYB21^1‐614^, PuMYB21^1‐891^, PuMYB21^292‐891^ and PuMYB21^592‐891^ (Figure 6A); yeast two‐hybrid (Y2H) analysis was then conducted to map the minimal interaction domain between PuMYB21 and PuERF106. Co‐transformation of PuMYB21^1‐891^, PuMYB21^292‐891^, or PuMYB21^592‐891^ with the empty AD vector resulted in yeast growth on both SD/‐Trp/‐Leu and SD/‐Trp/‐Leu/‐Ade/‐His media, demonstrating that these three N‐terminal truncations possess intrinsic transcriptional activation activity. For pairwise interaction tests, yeast harboring either PuMYB21^1‐336^ + PuERF106 or PuMYB21^1‐614^ + PuERF106 survived on double‐dropout medium. However, only yeast co‐expressing PuMYB21^1‐614^ and PuERF106 formed colonies on quadruple‐dropout medium, verifying that the 1‐614 amino acid segment of PuMYB21 directly mediated its association with PuERF106 (Figure 6B). The *in vivo* interaction between PuMYB21 and PuERF106 was confirmed by LCI assays, which showed strong luminescence only in co‐infiltrated samples. This interaction was significantly disrupted by exogenous H_2_S treatment (Figure 6C,D). It was hypothesized that H_2_S impairs complex assembly via protein persulfidation and subsequent conformational changes. Direct physical binding between PuMYB21 and PuERF106 was verified by *in vitro* pull‐down assays (Figure 6E). Structural modeling identified four conserved residue pairs that potentially mediate the interaction through hydrogen bond formation (Figure S8A–C).

### Persulfidation at Cys162 of PuERF106 attenuates its interaction with PuMYB21

Previous transcriptomic analysis revealed that exogenous H_2_S significantly repressed the transcript levels of *PuFAD8* and *PuERF106* (Figures 2E and 5B), and disrupted the physical interaction between PuERF106 and PuMYB21. Amino acid sequence alignment identified three conserved cysteine residues in PuMYB21, whereas only one was found in PuERF106 (Figure S7B). To elucidate the post‐translational regulatory mechanism underlying H_2_S‐modulated PuERF106‐PuMYB21 interaction, modified biotin‐switch assays were performed to detect protein persulfidation levels. Undetectable persulfidation modification was observed in PuMYB21, whereas robust H_2_S‐dependent persulfidation signals were identified in PuERF106 (Figure 6F; Figure S7C). To pinpoint the functional persulfidation site in PuERF106, we generated the PuERF106^C162A^ mutant via site‐directed mutagenesis (codon TGT → GCG). Persulfidation assays showed that this mutant exhibited no detectable modification changes under gradient H_2_S treatments (Figure 6G).

To determine how H_2_S‐induced persulfidation modulates the PuERF106‐PuMYB21 interaction, we performed Y2H assays. All transformants co‐expressing PuMYB21^1‐336^/PuERF106^C162A^ or PuMYB21^1‐614^/PuERF106^C162A^ grew normally on SD/‐Trp/‐Leu medium. On SD/‐Trp/‐Leu/‐Ade/‐His quadruple‐selective medium, only the PuMYB21^1‐614^‐PuERF106^C162A^ combination showed robust growth, demonstrating that the Cys162 point mutation did not completely abrogate their physical interaction (Figure 6H). Subsequent *in vitro* GST pull‐down assays quantified the effect of H_2_S on PuERF106‐PuMYB21 binding affinity. The interaction between PuERF106^C162A^ and PuMYB21 was retained but significantly attenuated. Exogenous H_2_S markedly reduced PuERF106‐PuMYB21 binding, and this inhibition was completely reversed by dithiothreitol (DTT), a disulfide bond reducer. Notably, the PuERF106^C162A^ variant showed a blunted response to H_2_S‐induced inhibition (Figure 6I). LCI assays were performed to validate the in planta effect of H_2_S on this interaction. No significant luminescence change was detected following H_2_S treatment in PuMYB21/PuERF106^C162A^ co‐infiltrated samples, but the interaction activity was markedly lower than that of the PuMYB21‐PuERF106 pair (Figure 6J,K). Consistent with *in vitro* observations, H_2_S treatment significantly attenuated the in planta interaction between PuERF106 and PuMYB21 but exerted no effect on the interaction between PuERF106^C162A^ and PuMYB21. DLR assays were then performed to examine whether PuERF106 persulfidation modulates downstream transcription by disrupting its association with PuMYB21. Relative to the wild‐type combination, co‐expression of PuERF106^C162A^ and PuMYB21 significantly reduced *PuFAD8* promoter transactivation, and no detectable change in the transactivation efficiency of this mutant complex was observed after H_2_S exposure (Figure 6L,M). Collectively, H_2_S‐mediated persulfidation of PuERF106 at Cys162 disrupts the PuERF106‐PuMYB21 heterodimer and represses *PuFAD8* transcriptional activation.

### The PuMYB21–PuERF106 transcriptional complex activates *PuFAD8* to modulate fatty acid‐derived volatile aroma biosynthesis

**Figure 7:**
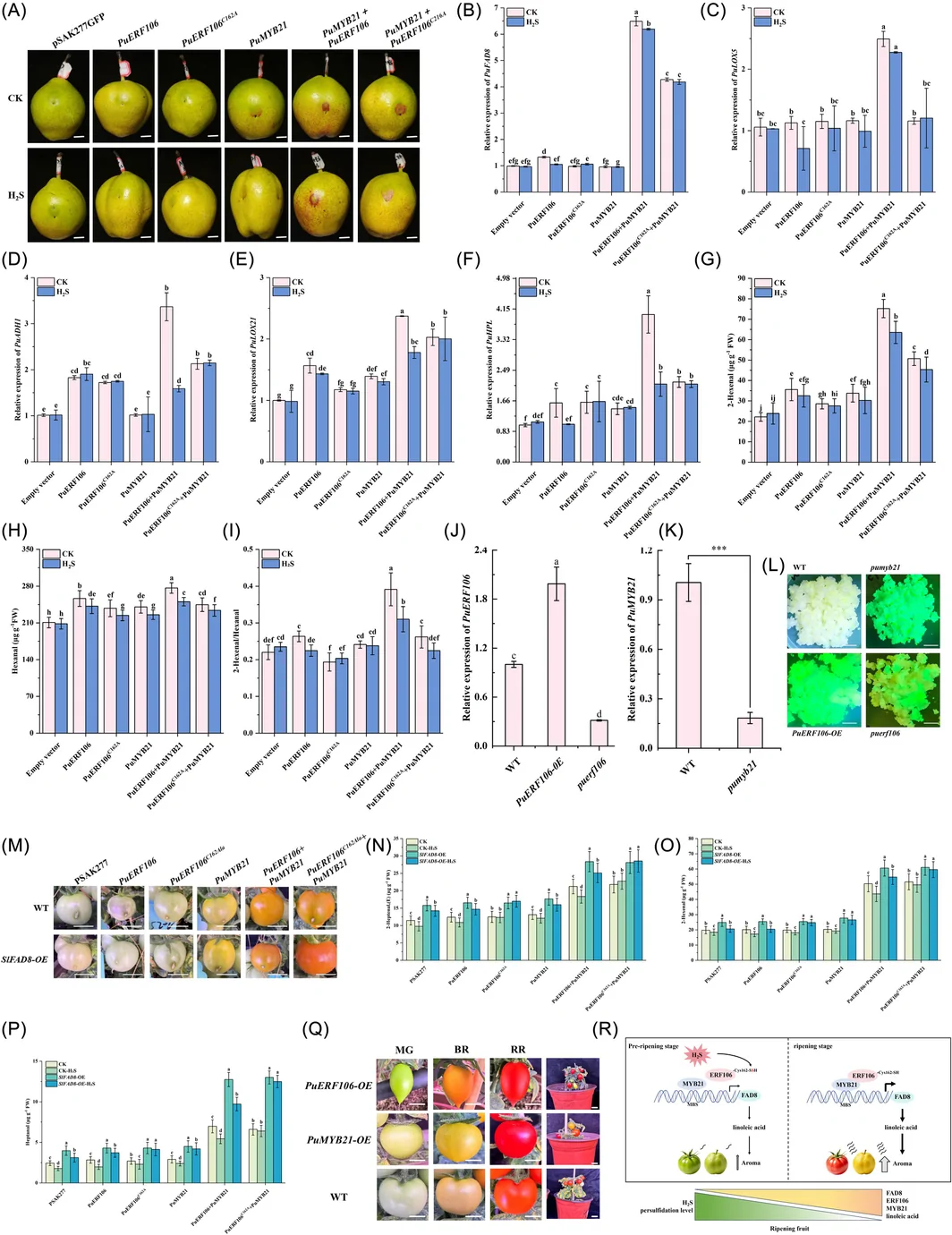
H_2_S‐mediated persulfidation of PuERF106 suppresses assembly of the PuERF106–PuMYB21 complex to modulate *PuFAD8*‐dependent aroma biosynthesis. (A) Phenotypic analysis of pear fruits transiently transformed with *PuMYB21*, *PuERF106*, or *PuERF106* ^*C162A*^. Scale bars = 1 cm. (B–F) RT‐qPCR analysis of transcript levels for *PuFAD8* (B); *PuLOX5* (C);* PuADH1* (D); *PuLOX21* (E); *PuHPL* (F) in transiently transformed pear fruits with or without H_2_S treatment. (G–I) Quantification of aroma volatile compounds in transiently transformed pear fruits with or without H_2_S treatment: (E)‐2‐Hexenal (G), Hexanal (H), and the (E)‐2‐Hexenal/Hexanal ratio (I). (J–L) *PuERF106‐OE*, *PuERF106‐c9*, and *PuMYB21‐c9* transgenic pear callus lines were successfully established. RT‐qPCR analysis of transcript levels for *PuERF106* (J) and *PuMYB21* (K). Phenotypes of transgenic pear callus lines of *PuERF106‐OE*, *PuERF106‐c9*, and *PuMYB21‐c9* were presented (L). Scale bars = 1 cm. (M) Phenotype of tomato fruits transiently overexpressing *PuFAD8*. Scale bars = 1 cm. (N–P) Quantification of aroma volatile compounds in transiently transformed pear fruits with or without H_2_S treatment: (E)‐2‐Hexenal (N), (E)‐2‐Heptenal (O), and Heptanal (P). (Q) Phenotypes of tomato lines heterologously overexpressing PuERF106 and PuMYB21 were presented. MG = mature green, BR = breaker, RR = red ripe. Scale bars = 1 cm. (R) Working model of H_2_S‐mediated persulfidation repressing aroma biosynthesis in ‘Nanguo’ pear fruits. At the early stage of fruit ripening under high H_2_S levels (left panel), persulfidation of PuERF106 at Cys162 disrupts its interaction with PuMYB21, thereby inhibiting the transcriptional activation of *PuFAD8* and weakening aroma biosynthesis. In contrast, under low H_2_S conditions (right panel), unmodified PuERF106 forms a functional complex with PuMYB21 to directly activate *PuFAD8* expression, thus promoting the accumulation of characteristic aroma compounds (Aroma). During fruit ripening, the gradual decrease in endogenous H_2_S concentration relieves this repression, ultimately enhancing fruit aroma and quality. Data are presented as mean ± standard deviation (SD) of three biological replicates. Different letters above bars indicate significant differences as determined by one‐way ANOVA followed by Duncan's multiple range test (*P* < 0.05).

Transient infiltration was applied to ‘Nanguo’ pear fruits to examine the functions of PuMYB21, PuERF106, and responses to H_2_S and the PuERF106^C162A^ mutation. Phenotypes of different treatment groups were presented in Figure 7(A). Transcript abundances of *PuFAD8*, *PuLOX5* and *PuLOX21* were barely altered by separate infiltration of the tested transcription factors. Marked upregulation of these genes was triggered by PuMYB21 and PuERF106 co‐expression. Moderately reduced gene expression was recorded in PuERF106^C162A^‐infiltrated fruits relative to the PuERF106 group. H_2_S treatment caused strong transcriptional repression in PuMYB21‐PuERF106 co‐expressing fruits, while such repression was absent in the PuERF106^C162A^‐PuMYB21 combination. Persulfidation at PuERF106 Cys162 was shown to impair the synergistic activity of the regulatory module and inhibit expression of core aroma synthetic genes (Figure 7B–F). Contents of (E)‐2‐Hexenal and Hexanal were slightly decreased in fruits with single transcription factor infiltration. Accumulation of these volatiles was significantly increased by *PuMYB21* and *PuERF106* co‐expression (Figure 7G,H). The (E)‐2‐Hexenal/Hexanal ratio was unaltered in single‐infiltration groups but greatly raised in co‐infiltrated fruits. Volatile levels and their ratio were obviously suppressed by H_2_S in PuMYB21‐PuERF106 co‐expressing fruits, while no comparable alterations were detected in PuERF106^C162A^‐PuMYB21 samples (Figure 7I). Stable pear transgenic lines are difficult to produce. Thus, only *PuERF106‐OE*, *PuERF106‐c9*, and *PuMYB21‐c9* lines were obtained, and their identity was confirmed via RT‐qPCR (Figure 7J–L).

Functional conservation of the PuMYB21‐PuERF106 module was assessed by transient infiltration in tomato. The ripening process was promoted by co‐expression of the two transcription factors (Figure 7M). Increased accumulation of target aroma compounds was observed in *SlFAD8‐OE* lines, and their synthesis was further strengthened by the heterologous complex (Figure 7N–P). Transgenic tomato lines overexpressing *PuERF106* and *PuMYB21* were produced to validate the regulatory complex (Figure 7Q). The homologous genes were identified using online bioinformatic databases (https://phytozome‐next.jgi.doe.gov/): *SlERF106* (*Solyc05g052030.1.1*, 49% sequence identity) and *SlMYB21* (*Solyc05T000420.1*, 82% sequence identity) (Figure S9A–D). All lines were confirmed heterozygous (Figure 7M; Figure S10A–D), and homozygous line selection is in progress.

## DISCUSSION

### H_2_S signaling modulates *PuFAD8* ‐dependent linoleic acid biosynthesis for volatile aroma production in ‘Nanguo’ pear

Fruit flavor represents a decisive commercial trait that profoundly shapes consumer acceptability and market value. The major volatile constituents responsible for fruit flavor mainly consist of esters, alcohols, aldehydes, and terpenoids (Luo et al., 2021; Yan et al., 2020). The ‘Nanguo’ pear is a typical climacteric fruit, coupled with the emission of abundant signature volatile aroma compounds (Zhou et al., 2015). Aromatic quality is therefore regarded as one of the most critical determinants of the overall commercial and sensory quality of ‘Nanguo’ pear fruits. The biosynthesis of volatile aroma compounds in pear fruits is primarily mediated by two major metabolic pathways: the LOX pathway and fatty acid β‐oxidation. In this sequential enzymatic cascade, unsaturated fatty acids are successively catalyzed by LOX, HPL, and ADH enzymes to generate volatile aldehydes and alcohols, which are further esterified by alcohol AAT to produce a diverse array of characteristic fruity esters. This ester‐dominant volatile mixture is ultimately responsible for the distinctive aromatic and flavor attributes of ‘Nanguo’ pear (Zhu et al., 2024). In the vast majority of fleshy fruit species, unsaturated fatty acids serve as essential primary precursors for the biosynthesis of volatile aroma compounds.

In pear fruits specifically, linoleic acid and α‐linolenic acid constitute the two predominant substrates for aroma volatile production. The fatty acid desaturase (FAD) enzyme family represents a critical set of rate‐limiting enzymes that directly control the biosynthesis and accumulation of these essential unsaturated fatty acid precursors (Tang et al., 2025). Previous evidence has demonstrated that FAD members, such as *PuFAD2* (Liu, Qin, et al., 2024) in ‘Nanguo’ pear, are essential for unsaturated fatty acid metabolism. In this study, exogenous H_2_S fumigation treatment remarkably suppressed the catalytic activities of LOX, HPL, and AAT enzymes in ‘Nanguo’ pear fruits, which was accompanied by a significant decrease in the endogenous levels of oleic acid, linoleic acid, and α‐linolenic acid (Figure 1). *PuFAD8* encodes a plastid‐localized ω‐3 fatty acid desaturase. To date, existing research on plant *FAD8* has largely focused on abiotic stress adaptation (Matsuda et al., 2005; Soria‐Garcï et al., 2019; Xu et al., 2023), whereas its regulatory roles in fruit ripening and volatile aroma metabolism remain poorly understood. Transcriptional detection via RT‐qPCR verified that *PuFAD8* is actively involved in fatty acid metabolism and aroma compound accumulation during the early ripening stage of ‘Nanguo’ pear (Figure 2). Given the technical limitations of stable genetic transformation in pear, we utilized tomato as a heterologous system for *in vivo* functional verification of PuFAD8 and its upstream regulatory module. This strategy is well supported by previous studies across multiple Rosaceae fruit crops, which have consistently demonstrated high functional conservation of core fruit quality regulatory pathways between tomato and perennial fruit trees. For instance, the ethylene biosynthetic gene *PyACS1* from pear (Sun, Cao, et al., 2025), the PpEIL2/3‐PpNAC1‐PpWRKY14 ripening regulatory module from peach (Liu, Xiao, et al., 2024), and the MdERDL6‐TST sugar accumulation module from apple (Zhu et al., 2021) have all been successfully validated in transgenic tomato systems, with heterologous expression producing physiological phenotypes fully consistent with their native functions. The fatty acid metabolic pathway underlying fruit aroma biosynthesis is deeply conserved across all climacteric fleshy fruits; thus, the tomato heterologous system provides a reliable and biologically relevant platform for characterizing the function of pear aroma‐related genes in this study. Accordingly, transient infiltration assays in pear fruit, combined with heterologous overexpression and genetic mutation analyses of *FAD8* homologs in tomato, confirmed that *PuFAD8* positively regulated aroma biosynthesis, and its biological function was tightly modulated by H_2_S signaling (Figures 3 and 4; Figure S4). Collectively, our findings demonstrate that H_2_S signaling acts as a key upstream regulatory module that precisely modulates *PuFAD8*‐mediated unsaturated fatty acid metabolism, which in turn governs the biosynthesis of postharvest aroma volatiles in ‘Nanguo’ pear fruits.

### *PuERF106‐PuMYB21* complex transduces H_2_S signals to regulate aroma biosynthesis via direct binding to the *PuFAD8* promoter

ERF and MYB transcription factors often work in tandem to govern plant metabolic pathways via the formation of heteromeric regulatory complexes. As an example, in strawberry, FaERF#9 and FaMYB98 form an ERF‐MYB transcriptional module that drives the production of 4‐hydroxy‐2,5‐dimethyl‐3(2H)‐furanone (HDMF), a prominent aroma volatile (Zhang et al., 2018). In petunia, PhERF6 interacts with the MYB‐like transcription factor EOBI to competitively modulate the production of phenylpropanoid volatiles (Liu et al., 2017). In apple, MdERF1B forms a complex with MdMYB1, MdMYB9, or MdMYB11 to synergistically drive anthocyanin biosynthesis (Sun et al., 2021). Similarly, a ternary complex consisting of ERF3‐MYB114‐bHLH3 has been implicated in regulating anthocyanin accumulation in red pear peel (Li, Qin, et al., 2025). Consistent with these findings, ERF and MYB members in pear have been linked to aroma formation; for example, PuERF008 modulates aroma precursor supply by regulating *PuFAD2* in the fatty acid metabolic cascade (Liu, Qin, et al., 2024). However, the mechanism by which ERF and MYB factors form complexes and integrate H_2_S signaling to govern aroma biosynthesis remains largely unexplored. Our experimental results revealed that exogenous H_2_S supply strongly downregulated the transcript levels of *PuFAD8* (Figure 2E). Meanwhile, modified biotin‐switch assays confirmed that the PuFAD8 protein did not undergo direct persulfidation modification (Figure S7A). This observation prompted us to hypothesize that additional upstream regulators may mediate H_2_S signaling toward *PuFAD8*. DLR assays revealed that co‐expression of *PuMYB21* and PuERF106 exerted the most prominent transactivation effect on the PuFAD8 promoter (Figure 5E). Previous studies have demonstrated that PyMYB10 interacts with PyMYB73 to form a regulatory complex that binds to MBS elements in the promoters of anthocyanin biosynthetic genes, thereby enhancing pigmentation in red‐skinned pears (Yao et al., 2023). Driven by this regulatory framework, we hypothesized that PuMYB21 and PuERF106 may form a functional complex to control *PuFAD8* expression and subsequent aroma volatile production. Y1H assays subsequently demonstrated that PuMYB21 directly bound to the *PuFAD8* promoter and activated its transcription, and this transactivation was markedly attenuated by H_2_S (Figure 5F,G). Protein–protein interaction assays further confirmed the physical association between PuERF106 and PuMYB21 (Figure 6). To validate the in planta function of the PuERF106‐PuMYB21 complex in modulating *PuFAD8*, we conducted transient transformation assays in pear fruit and functional rescue experiments in a *SlFAD8* transgenic tomato background (Figure 7). Collectively, these results establish that the PuERF106‐PuMYB21 complex serves as a key downstream effector of H_2_S signaling, directly activating *PuFAD8* transcription to orchestrate aroma compound biosynthesis in ‘Nanguo’ pear.

### H_2_S‐mediated persulfidation modification of PuERF106 at cysteine 162 disrupts aroma compound biosynthesis in pear fruits

Protein persulfidation represents a fundamental post‐translational regulatory mechanism that transduces H_2_S‐derived signaling events in plant systems. Through the covalent modification of reactive cysteine residues, H_2_S dynamically regulates protein three‐dimensional structure, protein–protein interaction networks, and enzymatic activities, which in turn orchestrate a diverse array of downstream physiological and metabolic processes. Accumulating evidence has demonstrated that fruit ripening and senescence processes are predominantly regulated by H_2_S‐mediated persulfidation of key transcriptional regulators. In planta, H_2_S governs fruit maturation and senescence predominantly via persulfidation of transcription factors. Accumulating evidence has illustrated the widespread biological functions of H_2_S‐mediated persulfidation across horticultural crops. In pear, persulfidation of PuMYB10 modulates anthocyanin accumulation in red‐skinned fruit (Yao et al., 2023). In tomato, H_2_S enhanced the transcriptional capacity of SlERF.D3 via cysteine modification to delay leaf senescence and fruit ripening (Hu, Geng, et al., 2025); H_2_S repressed the transcriptional activity of SlERF.D2 to antagonize ethylene synthesis and signaling, further slowing tomato ripening (Sun, Zhang, et al., 2025). Moreover, H_2_S disrupts the assembly of the KAT1‐KAT2 heteromeric channel through persulfidation at the Cys647 site of KAT1, suppressing inward K^+^ influx and facilitating stomatal closure (Liu et al., 2025). Nevertheless, whether H_2_S‐triggered transcription factor persulfidation controls volatile aroma metabolism in pear fruit remains largely uncharacterized. Herein, we demonstrated that the PuMYB21‐PuERF106 transcriptional complex acted as a core H_2_S signaling module to activate *PuFAD8* and modulate aroma biosynthesis. A modified biotin‐switch assay verified that PuMYB21 is insensitive to H_2_S‐induced persulfidation (Figure S7C). In contrast, PuERF106 contains only one conserved cysteine residue (Cys162), which serves as the exclusive target for H_2_S‐mediated persulfidation (Figure S7B). Furthermore, this cysteine residue is highly conserved across species, including tomato and *Arabidopsis thaliana* (Figure S11). Site‐directed mutagenesis further confirmed that mutation of Cys162 completely abolished the persulfidation signal of PuERF106 (Figure 6G). Furthermore, genetic alteration of this key residue markedly attenuated the physical interaction between PuERF106 and PuMYB21 (Figure 6H–M).

## MATERIALS AND METHODS

### Plant materials and growth conditions

*Pyrus ussuriensis* cv. ‘Nanguo’ pear fruits were harvested from a standardized experimental orchard and screened for uniformity. ‘Huangjin’ and ‘Qixia Daxiangshui’ pear fruits for comparative validation were harvested at matching commercial maturity from the same orchard.

*Micro‐Tom* tomato (*Solanum lycopersicum*) was used for heterologous functional validation. Plants were cultivated under controlled conditions in a greenhouse (23 ± 2°C; 50–70% relative humidity [RH] under conditions of 16 h light/8 h dark and 250 μmol m^−2^ sec^−1^ light).

*Nicotiana benthamiana* plants were grown at 25°C with a 16‐h photoperiod in a greenhouse at Hefei University of Technology.

### Isolation of callus tissue from genetically modified pear fruits

Pyrus communis callus was generated from surface‐sterilized explants and maintained through periodic subcultures on solid MS medium supplemented with 30 g L^−1^ sucrose, 0.5 mg L^−1^ 6‐BA and 1.0 mg L^−1^ 2,4‐D under dark conditions. Vigorous, axenic callus lines were chosen for Agrobacterium‐mediated genetic transformation. The transformation procedure was adapted from previous reports (Ming et al., 2022): Agrobacterium infection solution was pre‐incubated with gentle shaking in darkness for 1 h, followed by 30 min incubation with pear callus. Excess bacterial suspension was removed by blotting, and calli were co‐cultivated for 48 h in darkness. Transgenic calli were recovered on selective medium supplemented with 200 mg mL^−1^ kanamycin and 200 mg mL^−1^ timentin at 25°C, with subculturing performed biweekly. Primers used for recombinant plasmid construction were provided in Table S1.

### Enzyme activity assay

Lipoxygenase (LOX), alcohol dehydrogenase (ADH), hydroperoxide lyase (HPL), and alcohol acyltransferase (AAT) activities were determined in crude enzyme extracts following the method described by Lara et al. (2003). One unit (U) of enzyme activity was defined as a 1.0‐unit change in absorbance per minute under standard assay conditions. Crude enzyme extracts were prepared from three independent biological replicates of frozen fruit flesh. Approximately 1.0 g of tissue was ground to a fine powder in liquid nitrogen with 10% (w/w) insoluble polyvinylpyrrolidone (PVP) to prevent protein denaturation and oxidation. Four milliliters of pre‐chilled (4°C) extraction buffer was added, and the mixture was vortexed thoroughly to obtain a homogeneous extract. Enzyme activity was assayed by continuously monitoring absorbance changes at 234 nm (ΔOD_234_) for 1 min, starting 15 sec after adding the crude extract to the reaction mixture. Specific activity was calculated from the linear slope of ΔOD_234_ per minute and expressed as units per milligram of total soluble protein (mU mg^−1^ protein).

### Determination of fatty acid content by gas chromatography

Fatty acid profiling was carried out using an Agilent 7820 GC system fitted with a DB‐WAX capillary column and FID detector. Injector and FID temperatures were maintained at 250 and 280°C, respectively. Ultra‐high purity nitrogen served as the carrier gas with a steady flow rate of 1.0 mL min^−1^. The oven temperature was initially held at 50°C for 1 min, followed by a linear increase to 200°C at 25°C min^−1^, a further increase to 230°C at the same rate, and a final hold for 18 min. Samples (5 μL) were injected in split mode with a split ratio of 1:1. Fatty acid species were identified by matching retention times against a mixed standard containing 37 fatty acid methyl esters (C4–C24). Relative contents were calculated using the peak‐Sarea normalization method and were presented as a percentage of total fatty acids.

### GC–MS analysis of volatile aromatic compounds

Volatile metabolite profiling was performed according to the published method (Qin et al., 2012). An Agilent 7890A GC system interfaced with a 5975B mass selective detector was employed for compound separation and identification. Chromatographic separation was achieved on an HP‐5 MS capillary column (30 m × 0.25 mm × 0.25 μm). The oven temperature was initially held at 35°C for 5 min before programmed heating. Ultra‐high purity nitrogen served as the carrier gas with a constant linear velocity of 1.0 mL min^−1^. Temperatures of the injector, detector, and transfer line were maintained at 250, 270, and 280°C, respectively. Mass spectra were acquired in EI mode at 70 eV ionization energy, with operating parameters set as follows: filament current 0.25 mA, electron multiplier voltage 1724 V, scan range 33–350 amu, ion source temperature 230°C, quadrupole temperature 150°C. Volatile components were identified by mass spectral matching against the NIST 11.1 database, followed by manual confirmation with authentic standards. Relative contents of target compounds were calculated using the internal standard method, with 3‐nonanone (≥97.0%, GC grade) as the internal standard. A 0.04 g L^−1^ internal standard solution was prepared, and 50 μl of each sample was injected into the GC–MS system.

### Total RNA extraction and RT‐qPCR analysis

Total RNA isolation was performed on pear peel and flesh samples with the FastPure^®^ Universal Plant Total RNA Extraction Kit (Vazyme, Cat. No. RC411). Reverse transcription was carried out using PrimeScript™ RT Master Mix (Takara Bio) strictly following the manufacturer's protocol. RT‐qPCR amplifications were conducted with SYBR^®^ PreMix Ex Taq™ II (Takara Bio) under optimized reaction conditions. Gene‐specific primers were provided in Tables S2 and S3. The thermal profile consisted of an initial denaturation step at 94°C, followed by 40 cycles of 15 sec at 94°C, 15 sec at 60°C, and 20 sec at 72°C. Relative transcript abundances were determined using the comparative 2^−ΔΔCT^ method. Results were expressed as mean ± SD from three biological replicates.

### Analysis of FAD gene sequences across different species

Phylogenetic analysis was conducted using MEGA 7.0 with the neighbor‐joining clustering algorithm. The topological reliability of the phylogenetic tree was evaluated by bootstrap analysis with 1000 replicates. Sequence homology among FAD family members was calculated using DNAMAN software.

### Generation of stable transgenic overexpression and gene deletion tomato lines

To characterize the biological function of PuFAD8, its coding sequence was used as a query to retrieve tomato orthologs through NCBI BLAST. Amino acid sequence alignment revealed that *SlFAD8* (*Solyc08g063090.2.1*) exhibited 61% sequence identity with PuFAD8. The pCAMBIA1300‐*SlFAD8* construct was transformed into Micro‐Tom tomato via *Agrobacterium strain* EHA105, and T_2_‐generation stable overexpression lines were isolated for phenotypic and molecular assays. SlFAD8‐specific CRISPR guide RNAs were designed using CRISPRDirect. The double‐stranded target fragment was PCR‐amplified and inserted into the pYLCRISPR/Cas9‐DN vector via Golden Gate assembly (Engler & Marillonnet, 2013). This plant genome editing vector was developed by Professor Yaoguang Liu. Homozygous T_2_ *slfad8* mutant lines were recovered for further functional studies. *slmyb21* and *slerf106* lines were produced using an identical methodology. All cloning primers were provided in Table S4.

### VIGS and transient transformation in pear fruits

A 400 bp *PuFAD8*‐specific silencing fragment was designed and selected via the SGN VIGS web server. The amplified fragment was inserted into the pTRV2 vector, and the recombinant construct was transformed into *Agrobacterium tumefaciens* GV3101. VIGS experiments in tomato fruits were performed following the published protocol (Fantini & Giuliano, 2016).

For transient expression assays, full‐length CDSs of *PuERF106* (including the C162A mutant), *PuMYB21*, *PuFAD8*, and nine related transcription factors were cloned into the pSAK277 vector under the control of the CaMV 35S promoter using *Eco*RI and *Xba*I sites (Hellens et al., 2005). Positive constructs were introduced into *A. tumefaciens* GV3101. Bacterial cultures were adjusted to equal densities and mixed in equal volumes, and 300 μl aliquots were infiltrated into pear fruits. Infiltrated tissues were collected 7 days later, frozen in liquid nitrogen, and stored at −80°C. Primers used for vector construction were provided in Table S5.

### RNA extraction and transcriptome sequencing using the Illumina platform

As described previously (Zhong et al., 2021), tomato fruits were collected 20 days after flowering and randomly assigned to six experimental groups (12 fruits per group). Foliar treatments consisted of either 5000 mL distilled water (control) or 500 mL 0.75 mM NaHS (H_2_S donor), with three biological replicates per treatment. Fruit samples were harvested at 1 and 3 days post‐treatment, with untreated controls designated CK‐3d and H_2_S‐exposed samples designated H_2_S‐3d. Total RNA isolation was carried out using TRIzol reagent (Invitrogen) following standard protocols. RNA quality was evaluated via spectrophotometry (NanoDrop 2000, Thermo Scientific) and capillary electrophoresis (Agilent 2100 Bioanalyzer). Sequencing libraries were prepared using the VAHTS Universal V6 RNA‐seq Library Kit. Transcriptome sequencing and downstream computational analyses were conducted by OE Bio‐Technology Co., Ltd. (Shanghai, China).

### Dual‐luciferase reporter (DLR) assay

Effector constructs were generated by subcloning full‐length CDSs of PuERF106, eight additional ERF/RAP2 family members, PuMYB21, and PuMYB44 into the pSAK277 vector. The *PuFAD8* promoter region (2000 bp upstream of the translation start site) was ligated into the pGreenII 0800‐Luc vector to generate the reporter construct. All plasmids were co‐transformed into *A. tumefaciens* GV3101 with the pSoup helper plasmid at a molar ratio of 9:1, and verified positive strains were used for transient expression assays. Dual‐luciferase activities were measured 3 days after agroinfiltration using the Promega Steady‐Glo assay system. For exogenous H_2_S fumigation, tobacco leaf discs were incubated in airtight desiccators with 1 mM NaHS or distilled water for 2 h at 25°C under controlled humidity (70–80%). Treated samples were collected immediately for downstream analyses. Primer sequences for vector construction were provided in Table S6.

### Yeast one‐hybrid (Y1H) assay

Direct physical interaction between PuMYB21 and the *PuFAD8* promoter was investigated via Y1H analysis. The *PuFAD8* promoter was dissected into three overlapping fragments (S1: −420 to −49 bp; S2: −967 to −578 bp; S3: −1408 to −977 bp) according to *cis*‐regulatory element distribution. Each fragment was PCR‐amplified and ligated into the pAbAi bait plasmid, while the full‐length PuMYB21 CDS was subcloned into the pGADT7 prey vector. Bait constructs were sequentially transformed into Y1HGold–competent yeast cells. Positive interactions were identified by yeast growth on medium containing AbA, as PuMYB21 binding drives AbA resistance gene expression. Primer sequences for vector construction were provided in Table S6.

### Yeast two‐hybrid (Y2H) assay

The Matchmaker^®^ Gold Yeast Two‐Hybrid System (Clontech, http://www.clontech.com/) was employed to examine protein–protein interactions via yeast two‐hybrid (Y2H) assays. Five truncated fragments of PuMYB21, designated PuMYB21^1‐336^, PuMYB21^1‐614^, PuMYB21^1‐891^, PuMYB21^292‐891^, and PuMYB21^592‐891^, were separately cloned into the pGADT7 activation domain vector and the pGBKT7 DNA‐binding domain vector. The resulting recombinant constructs were co‐transformed into yeast strain AH109. Transformants were initially screened on SD/‐Leu/‐Trp dropout medium. Positive colonies were subsequently transferred onto stringent SD/‐Leu/‐Trp/‐His/‐Ade selective medium to verify protein–protein interactions. The combination of pGADT7‐T and pGBKT7‐Lam was used as the negative control, whereas pGADT7‐T paired with pGBKT7‐53 served as the positive control. All primers utilized for vector construction were listed in Table S7.

### Firefly luciferase complementation assay (FLC)

Firefly luciferase complementation (FLC) assays were conducted as described previously (Chen et al., 2008). Full‐length coding sequences of PuMYB21, PuERF106, and the PuERF106^C162A^ mutant (all devoid of termination codons) were separately subcloned into both the pCAMBIA1300‐NLuc and pCAMBIA1300‐CLuc dual reporter vectors. Agrobacterium‐mediated transient transformation of *N. benthamiana* leaves was performed as detailed in the preceding section. Firefly luciferase activity was measured at 72 h post‐infiltration using the Steady‐Glo^®^ Luciferase Assay Kit (Cat. No. E2510, Promega, USA). All oligonucleotide primers used for recombinant vector construction in this assay were provided in Table S8.

### In‐vitro pull‐down assay

GST pull‐down assays were performed to verify protein–protein interactions. Full‐length coding sequences (CDSs) of PuMYB21 and PuERF106 were individually subcloned into the pCOLD TF and pGEX4T‐1 expression vectors, respectively. All resultant recombinant plasmids were transformed into *Escherichia coli* Rosetta (DE3) competent cells, and recombinant protein expression was induced by 0.1 mM isopropyl‐β‐D‐thiogalactopyranoside (IPTG). Soluble GST‐tagged and GST‐fused PuERF106 proteins were purified and immobilized onto high‐capacity glutathione affinity resin. HIS‐tagged PuMYB21 (PuMYB21‐HIS) was then co‐incubated separately with immobilized GST (negative control) and PuERF106‐GST. Protein interactions were detected via Western blotting using an anti‐HIS monoclonal antibody. The PuERF106^C162A^ point mutant was similarly subcloned into pGEX4T‐1 to generate PuERF106^C162A^‐GST, and its interaction with PuMYB21 was examined using the identical protocol. Additionally, exogenous hydrogen sulfide (H_2_S) treatment was applied to investigate the regulatory effect of H_2_S on the PuMYB21‐PuERF106 physical interaction (Chen et al., 2020). All oligonucleotide primers used for vector construction were provided in Table S9.

### Detection of sulfhydryl modifications in PuMYB21 and PuERF106 recombinant proteins using the biotin switch method

Biotin‐switch assays were performed to evaluate the persulfidation levels of recombinant PuMYB21‐HIS and PuERF106‐GST proteins as described previously (Ma et al., 2021; Wedmann et al., 2016). Purified recombinant proteins were incubated with gradient concentrations of the hydrogen sulfide (H_2_S) donor NaHS (0, 400, and 800 μM). A parallel control group was treated with 1 mM dithiothreitol (DTT), a classical thiol‐reducing agent. Protein persulfidation was detected via Western blotting using a biotin‐conjugated primary antibody. To map the persulfidation site of PuERF106, site‐directed mutagenesis was conducted to substitute the cysteine codon (TGT) at residue 162 with an alanine codon (GCG). Mutagenesis was performed using the Mut Express II Rapid Mutagenesis Kit V2 (Vazyme Biotech Co., Ltd., Nanjing, China). The prokaryotic expression vector encoding PuERF106^C162A^‐GST was generated and sequence‐verified, and the persulfidation level of the resulting mutant fusion protein was examined using the identical biotin‐switch protocol.

### Statistical analysis

All assays were carried out with no fewer than three biological replicates. Mean values and standard deviations were calculated based on data obtained from three separate repeated trials. Significant differences among the parameter means (*P* < 0.05) were analyzed by Student's *t*‐test or Duncan's multiple tests. The data processing and graphics rendering were plotted by Origin Pro 9.0.

## ACCESSION NUMBERS

Sequence data from this article can be found in the genome database for the Rosa ceae (http://www.rosaceae.org), the National Center for Biotechnology Information (NCBI,https://www.ncbi.nlm.nih.gov/) or the Solanaceae (https://solgenomics.net/) under the following accession numbers: *PuFAD2* (*Pbr035830.1*), *PuFAD3* (*Pbr033732.1*), *PuFAD8* (*Pbr005591.1*), *PuHPL* (*Pbr041820.1*), *PuAAT* (*Pbr027302.1*), *PuLOX21* (*Pbr004967.1*), *PuLOX5* (*Pbr008100.1*), *PuADH* (*Pbr032983.1*), *PuPDC* (*Pbr016162.1*), *PuFATA* (*Pbr023307.1*), *PuSCD* (*Pbr005674.1*), *PuERF106* (*Pbr015699.1*), *PuERF9* (*Pbr016222.1*), *PuERF12* (*Pbr016224.1*), *PuABR1* (*Pbr019494.1*), *PuERF008* (*Pbr019669.1*), *PuERF110* (*Pbr024123.1*), *PuERF118* (*Pbr028836.1*), *PuERF4* (*Pbr030208.1*), *PuRAP2‐7* (*Pbr033937.1*), *PuRAP2‐4* (*Pbr007473.1*), *PuERF003* (*Pbr020024.1*), *PuRAP2‐3* (*Pbr012024.1*), *SlFAD2* (*Solyc01g006430.2*), *SlFAD3* (*Solyc06g007130.2*), *SlFAD8* (*Solyc08g063090.2*), *SlSCD* (*Solyc03g116730.2*), *SlFATA* (*Solyc06g083380.2*), *SlLOX21* (*Solyc01g006540.2*), *SlLOX5* (*Solyc09g075860.2*), *SlHPL* (*Solyc07g049690.2*), *SlAAT* (*Solyc07g049670.2*).

## AUTHOR CONTRIBUTIONS

Conceptualization: ZX, GY and HZ. Data curation: LY, HS, WZ and ZX. Formal analysis: LY, HS, WZ and ZX. Funding acquisition: GY, KH and HZ. Software: SW. Supervision: GY, KH, and HZ. Validation: ZX and GY. Writing—original draft: ZX and GY. Writing—review and editing: GY, KH, and HZ.

## CONFLICT OF INTEREST

The authors declare that they have no competing financial interests or personal relationships that could influence the work described in this paper.

## Supporting information


**Figure S1.** Aroma metabolic pathways and PCA analysis of volatile profiles in ‘Nanguo’ pear.
**Figure S2.** RT‐qPCR analysis of transcript levels for PuFADs and aroma biosynthesis‐related genes in ‘Nanguo’ pear fruit following H_2_S treatment.
**Figure S3.** *PuFAD8* computational bioinformatics analysis.
**Figure S4.** *PuFAD8* participates in the biosynthesis of fruit volatile aroma compounds.
**Figure S5.** Identification of a homozygous *slfad8* mutant line with a single base‐pair insertion.
**Figure S6.** Differential gene expression analysis in fruits of *SlFAD8‐OE*, *slfad8*, and wild‐type (WT) tomato plants under H_2_S treatment.
**Figure S7.** Persulfidation modification of target proteins and identification of their cysteine sites.
**Figure S8.** PyMOL docking model of the PuMYB21‐PuERF106 complex.
**Figure S9.** Spatiotemporal expression patterns of *PuMYB21* and *PuERF106* in pear and their orthologs *SlMYB21* and *SlERF106* in tomato.
**Figure S10.** Heterozygous mutant lines of *slerf106* and *slmyb21* were identified.
**Figure S11.** Multiple sequence alignment of *PuERF106* homologs from different plant species.

**Table S1.** Primer sequences for *PuERF106* and *PuMYB21* pear callus knockout and overexpression vector.
**Table S2.** Primer sequences for RT‐qPCR analyze the aroma‐related genes in pears.
**Table S3.** Primer sequences for RT‐qPCR of the aroma‐related genes in tomato fruits.
**Table S4.** All primers employed for the construction of recombinant vectors.
**Table S5.** The primers sequences for VIGS and transient expression experiments.
**Table S6.** The primers sequences for DLR and Y1H assay.
**Table S7.** The primer lists of *PuMYB21* and *PuERF106* for Y2H assay.
**Table S8.** The primer lists of *PuMYB21* and *PuERF106* for FLC assay.
**Table S9.** The primer lists of *PuMYB21* and *PuERF106* genes for conducting prokaryotic expression vector.

## Data Availability

The data that support the findings of this study are openly available in Pear Genomics Database at http://pyrusgdb.sdau.edu.cn/.
