# Supplementary material for: Hydrogen sulfide‐mediated persulfidation of ERF106 controls pear fruit aroma biosynthesis via disrupting the ERF106‐MYB21‐FAD8 complex
Source: Plant J. 2026 Sep 18;127(6):e71129. doi: 10.1111/tpj.71129 (PMC13588236; doi:10.1111/tpj.71129)
Supplement: Supplementary file 1 [file TPJ-127-0-s001.docx]

**Figure S1.** Aroma metabolic pathways and PCA analysis of volatile profiles in ‘Nanguo’ pear. **(A)** Metabolic pathways of aroma compounds in higher plants. **(B)** Principal component analysis (PCA) of major aroma constituent levels in ‘Nanguo’ pear between the H₂S‑fumigated treatment and control groups.

**Figure S2**. RT-qPCR analysis of transcript levels for PuFADs and aroma biosynthesis-related genes in ‘Nanguo’ pear fruit following H_2_S treatment. *PuFAD2* (A), *PuFAD3* (B). RT-qPCR analysis was performed to determine the transcript levels of PuFADs and aroma biosynthesis-related genes in ‘Huangjin’ pear and ‘Qixia Daxiangshui’ pear fruit. *PuFAD2* (C), *PuFAD3* (D).

**Figure S3.** *PuFAD8* Computational bioinformatics analysis. **(A)** Phylogenetic tree of FAD proteins across different plant species. **(B)** Conserved domain analysis.

**Figure S4.** *PuFAD8* participates in the biosynthesis of fruit volatile aroma compounds. **(A)** Phenotypic observation of pear fruit after transient overexpression of *PuFAD8*. Scale bars = 1 cm. **(B-F)** RT-qPCR analysis of the transcript levels of *PuFAD8* and key genes associated with aroma biosynthesis in *PuFAD8*-overexpressed fruits with or without H₂S treatment: *PuFAD8* **(B)**, *PuHPL* **(C)**, *PuLOX5* **(D)**, *PuAAT* **(E)**, and *PuADH1* **(F)**. **(G-K)** RT-qPCR detection of the expression patterns of *PuFAD8* and aroma biosynthesis-related genes in pear fruit with transient silencing of endogenous *PuFAD8*: *PuFAD8* **(G)**, *PuHPL* **(H)**, *PuLOX5* **(I)**, *PuAAT* **(J)**, and *PuADH1* **(K)**. All data in bar graphs are shown as mean ± standard deviation (SD), with at least three independent biological replicates. Statistical differences were determined by Student’s *t*-test: **p* < 0.05, ***p* < 0.01, ****p* < 0.001.

**Figure S5.** Identification of a homozygous *slfad8* mutant line with a single base‑pair insertion.

**Figure S6.** Differential gene expression analysis in fruits of *SlFAD8-OE*, *slfad8*, and wild-type (WT) tomato plants under H_2_S treatment. **(A)** *SlFAD8-OE*. **(B)** *slfad8*. **(C)** WT.

**Figure S7.** Persulfidation modification of target proteins and identification of their cysteine sites. **(A,B)** Persulfidation of recombinant PuFAD8/PuMYB21 proteins using a modified biotin switch assay. Recombinant proteins were incubated with NaHS (0, 400, and 800 μM) or 1 mM DTT, and persulfidated PuFAD8/PuMYB21 was detected by Western blot (the faint band in the figure corresponds to non-specific signal). PuFAD8**(A)**. PuMYB21**(B)**. **(C)** Cysteine sites in PuERF106/PuMYB21.

**Figure S8.** **(A-C)** PyMOL docking model of the PuMYB21 - PuERF106 complex. **(A)** Homology models of PuERF106. **(B)**Homology models of PuMYB21. **(C)** Predicted PuERF106 (cyan) and PuMYB21 (pink) docking model. The side chains of key residues from PuMYB21 (pink) and PuERF106 (cyan) are depicted as stick models. Yellow dashed lines denote noncovalent interactions (including hydrogen bonds and salt bridges) that mediate and stabilize the protein-protein complex. The core amino acid residues involved in the interaction (e.g., Y18, D8, K52, E206) are explicitly labeled, highlighting the critical molecular recognition interface between PuMYB21 and PuERF106.

**Figure S9.** Spatiotemporal expression patterns of *PuMYB21*, *PuERF106* in pear and their orthologs *SlMYB21*, *SlERF106* in tomato. **(A)** Spatio-temporal expression analysis of *PuMYB21* in pear fruit. **(B)** Spatio-temporal expression analysis of *PuERF106* in pear fruit. **(C)** Spatio-temporal expression analysis of *SlMYB21* in tomato plants. **(D)** Spatio-temporal expression analysis of *SlERF106* in tomato plants.

**Figure S10.** Heterozygous mutant lines of *slerf106* and *slmyb21* were identified. **(A, B)** *slerf106*. **(C, D)** *slmyb21.*

**Figure S11.** Multiple sequence alignment of *PuERF106* homologs from different plant species. Conserved cysteine residues are marked with dark blue boxes.
