# Supplementary material for: Hydrogen sulfide‐mediated persulfidation of ERF106 controls pear fruit aroma biosynthesis via disrupting the ERF106‐MYB21‐FAD8 complex
Source: Plant J. 2026 Sep 18;127(6):e71129. doi: 10.1111/tpj.71129 (PMC13588236; doi:10.1111/tpj.71129)
Supplement: Supplementary file 2 [file TPJ-127-0-s002.docx]

**Table S1.** Primer sequences for *PuERF106* and *PuMYB21* pear callus knockout and overexpression vector

| Primer name | Primer sequence |
| --- | --- |
| *PuERF106*-BsF | ATATATGGTCTCGATTGCTCATCCGCCAATACCTCTTGTT |
| *PuERF106*-F0 | TGCTCATCCGCCAATACCTCTTGTTTTAGAGCTAGAAATAGC |
| *PuERF106*-R0 | AACTGATTCAGAGAGGGACGAGTCAATCTCTTAGTCGACTCTAC |
| *PuERF106*-BsR | ATTATTGGTCTCGAAACTGATTCAGAGAGGGACGAGTCAA |
| *PuMYB21*-BsF | ATATATGGTCTCGATTGCGAACCATGGCGAAGGCCATGTT |
| *PuMYB21*-F0 | TGCGAACCATGGCGAAGGCCATGTTTTAGAGCTAGAAATAGC |
| *PuMYB21*-R0 | AACGTTACCCCACTTGGAGTGGACAATCTCTTAGTCGACTCTAC |
| *PuMYB21*-BsR | ATTATTGGTCTCGAAACGTTACCCCACTTGGAGTGGACAA |
| *PuERF106*-Pk7-F | GGGGACAAGTTTGTACAAAAAAGCAGGCTTCATGCCAGCAAATGAAGAACAAGA |
| *PuERF106*-Pk7-R | GGGGACCACTTTGTACAAGAAAGCTGGGTGTCAAGCACCTTCTCCTTCCCA |

**Table S2.** Primer sequences for RT-qPCR analyze the aroma-related genes in pears

| Gene name | Primer sequence |
| --- | --- |
| PuTubulin-F | GGGCTTTGCTCCTCTTACTT |
| PuTubulin-R | GATCAGCAGCACACATCATATTC |
| *PuFATA*-F | CTTCGCATACAAAGGGACA |
| *PuFATA*-R | CAGGTAACGGAACAAACG |
| *PuSCD*-F | TGCCACCGATGAGGAAGA |
| *PuSCD*-R | CGAGCCTAACCGCAACAA |
| *PuFAD2*-F | CCATTGCCTCCATCCTTTACT |
| *PuFAD2*-R | AGAACACAGCCCTGAACATAC |
| *PuFAD3*-F | AGAAGTCCAGGGAAGAAA |
| *PuFAD3*-R | CATCATAGTCCAGCAAGTAG |
| *PuFAD8*-F | AAGAGGAGCATCCCAGATAGA |
| *PuFAD8*-R | GCTCGCCAACACAAACATAAA |
| *PuLOX5*-F | GCAAGAGAGGAATGGCAGTT |
| *PuLOX5*-R | GTCCTGGAGTTCAAAATCACCT |
| *PuLOX21*-F | GACTACTTTCACCATCCCAGC |
| *PuLOX21*-R | CAACCCACTTGGTGTCTCTG |
| *PuHPL*-F | CGGTGTTCCGCACAAATA |
| *PuHPL*-R | AAGCGCAGGTCCTCAAGTC |
| *PuADH1*-F | TGCTGGTCAGGTCATACGC |
| *PuADH1*-R | CACGCCCTCACCAATACTC |
| *PuADH2*-F | GTAAGTCGGAGGAAAGCAA |
| *PuADH2*-R | AGATGCAATAGATGACCC |
| *PuAAT*-F | AAGGGCCTAACAGAATGC |
| *PuAAT*-R | CCTCCACAAGTAAGACGG |
| *PuERF106*-F | GGAGACTTCACCTTCACAGATT |
| *PuERF106*-R | ATTGAGGGCTGTGGTGATTAG |
| *PuERF105*-F | GCTTACGACTTTGCTGCTTTC |
| *PuERF105*-R | GATGATCGGCTGAGAGTTCTTC |
| *PuERF110*-F | CGGTGGAGTTACGTCTAGTTTC |
| *PuERF110*-R | GAAGCAGGAGAAGCACTTGA |
| *PuERF12*-F | AGTTGGTGTAGCGGGAAATG |
| *PuERF12*-R | TCGGACCAGTCCCAGAAA |
| *PuERF1*-F | GGTACCAACCACACCATCAA |
| *PuERF1*-R | CGTATCTCAGCTGCCCATTTA |
| *PuERF008*-F | CTGCTCTTCGTCGTCTTCTTC |
| *PuERF008*-R | CCTCATCCGTATTCCTCTGTATTG |
| *PuERF118*-F | GATTCCGACTCTGTCGTTTCTC |
| *PuERF118*-R | GGCAGTCTCCTTCTCCAAATC |
| *PuRAP2-3-*F | GAGTTTCCAGACCGGTTCTTAC |
| *PuRAP2-3*-R | GAACGACTCCAAGCTCCATATC |

**Table S3.** Primer sequences for RT-qPCR of the aroma-related genes in tomato fruits

| Gene name | Primer sequence |
| --- | --- |
| SL-Tubulin-F | TAGAGCCTGGTACGATGGATAG |
| SL-Tubulin-R | CAACTCAGCGCCTTCAGTATAA |
| *SlFAD8*-F | GTTGCATTTCATCCTGCTACTG |
| *SlFAD8*-R | GAACTCAGACACAAGCCTTCTA |
| *SlFAD2*-F | ACTTCCACCTCATTCCATCAC |
| *SlFAD2*-R | CGTGGGCATTAACCCAAATTC |
| *SlFAD3*-F | GCCCTATCATGGATGGAGAATTAG |
| *SlFAD3*-R | CATCGGCACCCAAGATTCA |
| *SlSCD*-F | CACTGGACTTCTCGGTATTACAC |
| *SlSCD*-R | TGCTTGGACACCACAATAGG |
| *SlADH*-F | TGTGCCACACTGATGTTTAC |
| *SlADH*-R | CACCTACACTCTCCACAATTCC |
| *SlLOX21*-F | GAGCTAACTAGGCCACCAATAG |
| *SlLOX21*-R | CCGGAGTCATGAGAAAGAACA |
| *SlHPL*-F | CATGGGTGCCTACACTACTTA |
| *SlHPL*-R | TTTGGAGTGCAGGAAGAAGAG |
| *SlAAT*-F | GGAAGGAGTTATGTTCGTGGAG |
| *SL-AAT*-R | GCTCTTCCAAACACGGAAATG |
| *SlFATA*-F | GACTATGTCGGAGGATGGAATG |
| *SlFATA*-R | CTGCAAGAGATTGGCGATAGT |

**Table S4.** All primers employed for the construction of recombinant vectors

| Primers | Sequence |
| --- | --- |
| *SlFAD8*-L177-F | TGTGTGTGCAGCCCGGGATCCATGAGTGATGATAAAAAGTACATTACTGCT |
| *SlFAD8*-L177-R | CATCAATTGACGCGTGTCGACTCCATGAGTATTAACAGCTTCCCA |
| *slfad8*-Target 1-F | ATTCAGGGCATTATCAGGT |
| *slfad8*-Target 1-R | GAGGTCTTTCACAAAGGGA |
| *slfad8*-Target 2-F | TTTCACAGTGACTGGAATTC |
| *slfad8*-Target 2-R | GCCGTAAGTAAGCCTTAATAGAA |
| *PuERF106*-L177-F | TGTGTGTGCAGCCCGGGATCCATGCCAGCAAATGAAGAACAAGA |
| *PuERF106*-L177-R | CATCAATTGACGCGTGTCGACAGCACCTTCTCCTTCCCAAAG |
| *slerf106*-Target 1-F | GTCAGGGTGAATCTGAGAGGGAAT |
| *slerf106*-Target 1-R | AAACATTCCCTCTCAGATTCACCC |
| *slerf106*-Target 2-F | GTCAGCTGTCGAAAACTGGACTG |
| *slerf106*-Target 2-R | AAACCAGTCCAGTTTTCGACAGC |
| *PuMYB21*-L177-F | TGTGTGTGCAGCCCGGGATCCATGGCTGCTCCTAGAAACCCTA |
| *PuMYB21*-L177-R | CATCAATTGACGCGTGTCGACCATCCCGTCCATGTTCCATAA |
| *slmyb21*-Target 1-F | ATTGCTACTCATGGCGAAGGTCGT |
| *slmyb21*-Target 1-R | AAACACGACCTTCGCCATGAGTAG |
| *slmyb21*-Target 2-F | ATTGCAAGAACTACTGGAGAACGA |
| *slmyb21*-Target 2-R | AAACTCGTTCTCCAGTAGTTCTTG |

**Table S5.** The primers sequences for VIGS and transient expression experiments

| Construct | Primer name | Primer sequence |
| --- | --- | --- |
| pSAK277-*PuERF106* | F | ACTAGTGGATCCAAAGAATTCATGCCAGCAAATGAAGAACAAGA |
|  | R | TCATTAAAGCAGGACTCTAGATCAAGCACCTTCTCCTTCCCA |
| pSAK277-*PuMYB21* | F | ACTAGTGGATCCAAAGAATTCATGGCTGCTCCTAGAAACCCTA |
|  | R | TCATTAAAGCAGGACTCTAGATCACATCCCGTCCATGTTCC |
| pSAK277-*PuFAD8* | F | ACTAGTGGATCCAAAGAATTCATGGAGGGTGAGAAGAAGTACATTACT |
|  | R | CATTCTAGAAGTACTCTCGAGACCATGGGTATTGACAGCTTCC |
| pSAK277-*PuERF105* | F | ACTAGTGGATCCAAAGAATTCATGGCATCAGAAGACTCTTCAGC |
|  | R | TCATTAAAGCAGGACTCTAGATCAAGTAACCATGAGCTGAGAATATCC |
| pSAK277-*PuERF110* | F | ACTAGTGGATCCAAAGAATTCATGTTTTTGGGGTACAGTCGGG |
|  | R | TCATTAAAGCAGGACTCTAGATCAACTGGATGAGGATGGATTGT |
| pSAK277-*PuERF12* | F | ACTAGTGGATCCAAAGAATTCATGGCGACTGCAGCTGCTC |
|  | R | TCATTAAAGCAGGACTCTAGATCACAGCCACATGGGAGGAG |
| pSAK277-*PuERF1* | F | ACTAGTGGATCCAAAGAATTCATGTGCTTACTGAAGGTGGCG |
|  | R | TCATTAAAGCAGGACTCTAGATCAACCGGATGAGGATGGATT |
| pSAK277-*PuERF008* | F | ACTAGTGGATCCAAAGAATTCATGGAAGGCGATTACTGCTGC |
|  | R | TCATTAAAGCAGGACTCTAGATTAACTTTCATCGGAGTTTTCTGG |
| pSAK277-*PuERF118* | F | ACTAGTGGATCCAAAGAATTCATGGCTGTGCCTCGGAAAC |
|  | R | TCATTAAAGCAGGACTCTAGATTATTGGCATACTATGTTGAGGGG |
| pSAK277-*PuRAP2-3* | F | ACTAGTGGATCCAAAGAATTCATGTGTGGTGGTGCTATCATTTCC |
|  | R | TCATTAAAGCAGGACTCTAGATTAATACAGAAGCTGCCCTTGTTG |
| TRV-*PuFAD8* | F | GTGAGTAAGGTTACCGAATTCACCATCCTCCATCCGCGT |
|  | R | CGTGAGCTCGGTACCGGATCCTGAGTCCATTTCCACCAAGCA |

**Table S6.** The primers sequences for DLR and Y1H assay

| Gene name | Direction | Primer sequence |
| --- | --- | --- |
| pAbAi-*Pu*FAD8-1 | F | AAATGATGAATTGAAAAGCTTCTTGAGCCGATCACGGATGT |
|  | R | ATACAGAGCACATGCCTCGAGCACTACTCATCTCCTTCCAAAATGAA |
| pAbAi-*Pu*FAD8-3 | F | AAATGATGAATTGAAAAGCTTTCCTAACCCAATTGGAGTCCC |
|  | R | ATACAGAGCACATGCCTCGAGCGAAGACCAGAACTTGAGTCTGG |
| pAbAi-*Pu*FAD8-3 | F | AAATGATGAATTGAAAAGCTTGTGGGTTCAAATCCCACTTCTG |
|  | R | ATACAGAGCACATGCCTCGAGGTCCTAGGTGTACAATGAATCGGA |
| AD-*PuMYB21* | F | GCCATGGAGGCCAGTGAATTCATGGCTGCTCCTAGAAACCCTA |
|  | R | ACGATTCATCTGCAGCTCGAGTCACATCCCGTCCATGTTCC |
| AD-*Pu*ERF106 | F | GCCATGGAGGCCAGTGAATTCATGCCAGCAAATGAAGAACAAGA |
|  | R | ACGATTCATCTGCAGCTCGAGTCAAGCACCTTCTCCTTCCCA |
| 0800-*PuFAD8* | F | GTCGACGGTATCGATAAGCTTGCGAGTTTATTTTCTAAAAGATCAACC |
|  | R | CGCTCTAGAACTAGTGGATCCAGCACACTATGTTGAAAATTACATGG |

**Table S7.** The primer lists of *PuMYB21* and *PuERF106* for Y2H assay

| Gene name | Primer sequence |
| --- | --- |
| BK-*PuMYB21*（1-336）-F | tcagaggaggacctgcatatgATGGCTGCTCCTAGAAACCCTA |
| BK-*PuMYB21*（1-336）-R | ctagttatgcggccgctgcagCCTCGTTCTCCAGTAGTTCTTTATCTC |
| BK-*PuMYB21*（1-614）-F | tcagaggaggacctgcatatgATGGCTGCTCCTAGAAACCCTA |
| BK-*PuMYB21*（1-614）-R | ctagttatgcggccgctgcagTTACTTGGGACGGTGGCAAG |
| BK-*PuMYB21*（1-891）-F | tcagaggaggacctgcatatgATGGCTGCTCCTAGAAACCCTA |
| BK-*PuMYB21*（1-891）-R | ctagttatgcggccgctgcagTCACATCCCGTCCATGTTCC |
| BK-*PuMYB21*（292-891）-F | tcagaggaggacctgcatatgCCGGGGAGAACAGACAATGA |
| BK-*PuMYB21*（292-891）-R | ctagttatgcggccgctgcagTCACATCCCGTCCATGTTCC |
| BK-*PuMYB21*（592-891）-F | tcagaggaggacctgcatatgAATCTTGCCACCGTCCCAA |
| BK-*PuMYB21*（592-891）-R | ctagttatgcggccgctgcagTCACATCCCGTCCATGTTCC |
| AD-*PuERF106*-F | gccatggaggccagtgaattcATGCCAGCAAATGAAGAACAAGA |
| AD-*PuERF106*-R | acgattcatctgcagctcgagTCAAGCACCTTCTCCTTCCCA |

**Table S8.** The primer lists of *PuMYB21* and *PuERF106* for FLC assay

| Gene name | Primer sequence |
| --- | --- |
| CLuc-*PuERF106*-F | CGAGCTCGGTACCCGGGATCCATGCCAGCAAATGAAGAACA |
| CLuc-*PuERF106*-R | CGCGTACGAGATCTGGTCGACTTCTCCTTCCCAAAGCACCC |
| NLuc-*PuMYB21*-F | CGAGCTCGGTACCCGGGATCCATGGCTGCTCCTAGAAACC |
| NLuc-*PuMYB21*-R | CGCGTACGAGATCTGGTCGACCATCCCGTCCATGTTCCAT |

**Table S9.** The primer lists of *PuMYB21* and *PuERF106* genes for conducting prokaryotic expression vector

| Gene name | Primer sequence |
| --- | --- |
| pCold-*PuMYB21*-F | CTCGGTACCCTCGAGGGATCCATGGCTGCTCCTAGAAACCCTA |
| pCold-*PuMYB21*-R | AGCAGAGATTACCTATCTAGATCACATCCCGTCCATGTTCC |
| pGEX-*PuERF106*-F | ccgcgtggatccccggaattcATGCCAGCAAATGAAGAACAAGA |
| pGEX*-PuERF106*-R | gtcacgatgcggccgctcgagTCAAGCACCTTCTCCTTCCCA |
